# Admission Prognostic Scores and Endoscopic Findings in Relation to the Two Components of Five-Day Treatment Failure in Acute Variceal Bleeding: An Exploratory Single-Centre Retrospective Cohort Study

**DOI:** 10.3390/diagnostics16213620

**Published:** 2026-11-02

**Authors:** Anca Monica Oprescu Macovei, Dana Paula Venter, Mihai Stefan, Constantin Oprescu, Andrei Gabriel Nicolae, Bogdan Dumitriu, Bianca-Maria Micle, Sebastian Valcea, Oana-Mihaela Plotogea, Madalina Ilie

**Affiliations:** 1 Faculty of General Medicine, Carol Davila University of Medicine and Pharmacy, 050474 Bucharest, Romaniamihai.stefan@drd.umfcd.ro (M.S.);; 2 Gastroenterology Department, Emergency Clinical Hospital Agrippa Ionescu, 011356 Bucharest, Romania; 3 General Surgery Department, Emergency Clinical Hospital Floreasca, 014461 Bucharest, Romania; 4 Gastroenterology Department, Emergency Clinical Hospital Floreasca, 014461 Bucharest, Romania

**Keywords:** acute variceal bleeding, liver cirrhosis, prognostic scores, Child–Pugh score, MELD, risk stratification, endoscopy, red wale markings, portal vein thrombosis

## Abstract

**Background/Objectives**: Five-day treatment failure after acute variceal bleeding combines failure of bleeding control and early rebleeding, which may reflect different clinical processes. We explored whether admission scores and endoscopic findings relate similarly to these components. **Methods**: All 303 consecutive admissions treated with band ligation at a tertiary centre during 2021–2025 were analysed. Six scores were compared using paired AUROCs and Holm adjustment. **Results**: Failure occurred in 31 admissions (10.2%): 15 failures of primary haemostasis and 17 rebleeds, with one admission meeting both components. Point estimates of discrimination were higher for rebleeding for all six scores (differences 0.123–0.236), but intervals were wide, and no formal between-component test was performed. No comparison among the six scores using the primary score definitions survived the Holm adjustment for the composite or either component; this does not establish equivalence. Active bleeding at endoscopy was associated with failure of primary haemostasis (odds ratio 4.52, 95% CI 1.40–14.53), whereas its association with rebleeding was uncertain (1.07, 0.40–2.90). Red wale markings showed no clear association. **Conclusions**: This exploratory pattern suggests that the components may capture different clinical processes, a hypothesis requiring prospective confirmation in a larger cohort. Non-standardised outcome classification, uncertain follow-up after transfer, intervening TIPS and sensitivity to recorded versus recomputed scores limit inference.

## 1. Introduction

Acute variceal bleeding is among the most severe complications of cirrhosis, and the population at risk is not diminishing. Alcohol-associated liver disease accounted for the loss of eleven million life-years worldwide in 2019, and the deaths attributed to it continue to rise [1]; in the same year alcohol was associated with approximately a quarter of all cirrhosis deaths [2]. Between 1990 and 2021 the global age-standardised mortality from alcohol-related cirrhosis fell from 5.40 to 4.08 per 100,000, but the decline was concentrated in the more developed countries, while mortality rose in Central and Eastern Europe and Central Asia [3]. Tertiary centres in this region therefore treat a case-mix that differs from the Western cohorts in which most prognostic tools were developed.

The management of the index bleed is standardised. Baveno VII [4], the ESGE guideline [5] and the AASLD guidance [6] agree on early vasoactive therapy, antibiotic prophylaxis, restrictive transfusion and band ligation within twelve hours, and Baveno VII also supplies the yardstick against which that package, and every prognostic score applied alongside it, is judged: failure within five days [4]. Standardisation has not abolished failure. Liu et al. reported five-day treatment failure in 16.7% of patients without hepatocellular carcinoma and in 21.1% of those with it [7], and Vasilakis et al., across 1864 ligation procedures, found a 25% five-day rebleeding rate in the subgroup that developed ligation-induced ulcer bleeding [8].

Five-day failure is a composite, but its operational definition matters when the components are examined separately. In this dataset, failure of primary haemostasis denotes failure of technical bleeding control at the index endoscopy. This is narrower than the broader clinical concept of failure to control bleeding over an acute episode, which also considers the subsequent clinical course [4]. Rebleeding is intended to denote recurrence after initial control, although that sequence could not be independently adjudicated in every record. Endoscopic accessibility, the bleeding state during treatment, portal pressure and hepatic reserve may contribute differently to these events, but their contributions cannot be inferred from timing alone. Combining components with different predictor associations can attenuate discrimination for the composite, although the magnitude and direction depend on the component frequencies, their overlap and the associations themselves.

Against this composite, the admission scores themselves have given inconsistent results. In 354 patients from five US centres, Buckholz et al. obtained almost identical discrimination for MELD, MELD 3.0 and Child–Pugh [9]; Kırdar et al. found the Child–Pugh and MELD scores more accurate than newer non-invasive fibrosis indices [10]; Demiröz et al. found measures of hepatic reserve superior to inflammatory markers [11]. In most of these series the scores are presented side by side without formal statistical comparison. A parallel literature substitutes systemic organ failure for hepatic reserve, with results that depend substantially on the setting in which patients were recruited [12,13]. A further source of information is generated at the index endoscopy and enters none of the scores: red wale markings are recorded in every endoscopy report [5] and formed part of the North Italian Endoscopic Club index for predicting a first variceal haemorrhage [14], and Juncu et al., in 217 patients submitted to a first emergency ligation, separated the predictors of variceal rebleeding from those of post-banding ulcer bleeding [15].

The older literature is consistent with the possibility without having tested it. Studies that examined five-day failure directly produced lists of independent predictors in which the two kinds of variable sit side by side: active bleeding at endoscopy in 695 consecutive admissions [16]; active bleeding, haematocrit, aminotransferase, Child–Pugh class and portal vein thrombosis in a large prospective series [17]; Child–Pugh class C, the white cell count and portal vein thrombosis in 185 unselected patients treated with the current standard [18]. These mixed predictor lists are compatible with several explanations, including differences between component associations, but they do not demonstrate such differences.

Taken together, these observations point to a gap rather than a resolution. Buckholz et al. [9], Kırdar et al. [10] and Demiröz et al. [11] compared admission scores against the composite five-day outcome or against longer-term mortality, but none asked whether an individual score discriminates differently for the two events that make up early failure. The older cohorts of Ben-Ari et al. [16], D’Amico and De Franchis [17] and Amitrano et al. [18] identified independent predictors of the composite endpoint that mix endoscopic and imaging findings, such as active bleeding and portal vein thrombosis, with measures of hepatic reserve, such as the Child–Pugh class; this pattern is consistent with the two components having different determinants but does not test that possibility directly, since the composite was never separated. We are not aware of a study that has decomposed the five-day endpoint and examined this question directly. This gap motivates the present analysis.

This study therefore asked, in a consecutive cohort treated with band ligation, whether admission scores and endoscopic findings relate similarly to the two components of the five-day endpoint. Three questions follow. Whether the six admission scores discriminate differently for failure of primary haemostasis and for rebleeding. Whether differences between scores can be demonstrated for the composite using paired comparisons with multiplicity adjustment. And whether red wale markings, a sign derived for the prediction of a first bleed, retain prognostic value once bleeding has occurred and has been treated. The study was not designed for the first question, which is a decomposition of an endpoint already recorded, so the component findings are interpreted descriptively and as hypothesis-generating.

## 2. Materials and Methods

### 2.1. Patients and Setting

This was a retrospective observational cohort study conducted at the Bucharest Emergency Clinical Hospital, a tertiary emergency referral centre. All consecutive adults admitted with acute variceal bleeding and treated with endoscopic band ligation between 8 January 2021 and 26 December 2025 were identified from the departmental endoscopy register and cross-checked against hospital discharge coding. Eligibility required established cirrhosis, oesophageal varices as the confirmed source at index endoscopy, graded F1 to F3, and band ligation. No patient was excluded on grounds of age, aetiology or comorbidity. The cohort comprises 303 admissions. Each record carries a unique study identifier, but that identifier is a sequential record number rather than a patient identifier, so the dataset does not allow repeat admissions by the same individual to be detected; the observations are treated as independent, although the number of distinct patients may be lower than 303 and dependence between their admissions cannot be quantified; the consequence is examined in Section 4.7. Transjugular intrahepatic portosystemic shunting is not available at the study centre. Patients in whom pre-emptive or rescue shunting was indicated were transferred to hospitals and clinics offering the procedure, and the database records whether the shunt was subsequently placed. Reporting follows the STROBE statement for observational studies [19] (Table S1) and, for the prediction-model component, the TRIPOD statement [20] (Table S2).

### 2.2. Data and Quality Control

Clinical, laboratory, endoscopic and outcome variables were abstracted from the hospital record into a structured database. Outcome classification is described separately in Section 2.4. Admission values were those recorded before any therapeutic intervention. Every continuous variable was screened against fixed biological ranges before analysis. Hepatic encephalopathy not described in the record was coded as absent. Long-term vital status was not ascertained. A substantial part of this cohort was referred from other counties and returned there after discharge. The study had no verified linkage to post-discharge vital-status records, so longer-term mortality was not analysed. Death within the five-day window was considered only in the sensitivity analysis described in Section 2.4 and Section 3.7.

### 2.3. Scores

Five scores were recomputed from the extracted admission components; ACLF grade was retained as recorded. MELD used bilirubin, INR and creatinine, with values below 1 set to 1, creatinine capped at 4 mg/dL, and the result rounded and bounded to 6–40. MELD-Sodium used the UNOS formula when MELD exceeded 11, with sodium bounded to 125–137 mmol/L. MELD 3.0 included sex, albumin and both interaction terms, with creatinine bounded to 1–3 mg/dL, albumin to 1.5–3.5 g/dL and sodium to 125–137 mmol/L, and was rounded and bounded to 6–40. Dialysis-specific creatinine overrides could not be verified because dialysis was not recorded. Child–Pugh used the conventional laboratory thresholds. The recorded ascites categories absent, mild-to-moderate and tense were mapped to 1, 2 and 3 points; encephalopathy grades 0, 1–2 and 3–4 were mapped to 1, 2 and 3 points. The clinical categories could not be independently re-adjudicated, including diuretic responsiveness of ascites. AIMS65 used albumin below 3.0 g/dL, INR above 1.5, encephalopathy grade 2 or higher as a proxy for altered mental status, systolic pressure of 90 mmHg or less and age above 65 years. A score was missing when a required component was unavailable. ACLF grade was abstracted as the admission classification recorded according to CLIF-C criteria. Respiratory and circulatory components and a verifiable assessment timestamp were unavailable, so neither the classification nor its timing could be independently validated. Comparisons involving ACLF therefore have a different evidential basis from those involving recomputed scores. The database also contained recorded Child–Pugh and MELD-Sodium values. Recorded Child–Pugh agreed exactly with recomputation in 240 of 277 comparable admissions and within one point in 266. Recorded MELD-Sodium agreed within one point in 215 of 281 and differed by up to eight points. Different clinical grading or formula versions are possible explanations, but the source of disagreement could not be established. Range screening and formula checks establish internal plausibility and reproducibility, not independent verification of every source measurement. Recorded and recomputed values were compared on identical available cases for the composite and both components, with additional recorded-score comparisons against MELD (Section 3.8; Table A4 and Table A5).

### 2.4. Outcomes

The primary outcome was recorded treatment failure within 120 h of the first assessment at the study centre, comprising failure of technical haemostasis at the index endoscopy and clinically significant rebleeding. For referrals from another hospital, time zero was the first assessment at this centre, not arrival at the referring hospital; the resulting window may therefore be shifted relative to the initial presentation. The first component was derived from the technical haemostasis field and the index endoscopy report and associated clinical notes. It should not be interpreted as a complete reconstruction of all consensus criteria for failure to control bleeding [4]. The rebleeding classification was abstracted from clinical and nursing notes, transfusion records, serial haemoglobin measurements and repeat-endoscopy reports when available. It included recurrent upper gastrointestinal bleeding recorded as clinically significant, without restriction to an oesophageal-variceal source. Post-banding ulcer bleeding was recorded when repeat endoscopy identified a ligation-site ulcer as the source without bleeding from a varix; events within 120 h also contributed to rebleeding. Secondary outcomes were rescue treatment during the index admission and post-banding ulcer bleeding. The detailed thresholds used in routine clinical classification were not documented in a written departmental protocol and could not be reconstructed consistently. The endpoint is therefore a pragmatic recorded outcome, not a retrospectively adjudicated Baveno endpoint. Outcome abstraction was not blinded to admission variables, and recorded Child–Pugh and MELD-Sodium values were available in the same records; recomputation of the five scores followed abstraction. One admission had both failed technical haemostasis and a rebleed recorded on day 4, with urgent repeat banding. It contributed once to the composite and once to each component. The dataset does not quantify the intervening period of bleeding control, so this sequence cannot be independently adjudicated; an analysis excluding this admission is reported in Section 3.8. Five deaths were recorded within 120 h, four after a failure event and one in an admission otherwise classified as a non-event. Death was not a separate component of the primary endpoint; adding it to the composite was examined in Section 3.7.

### 2.5. Statistical Analysis

Continuous variables are reported as median with interquartile ranges and compared by the Mann–Whitney U test; categorical variables are reported as counts and percentages and compared by Fisher’s exact test. Proportions have Wilson confidence intervals. Discrimination is summarised by the area under the ROC curve (AUROC). Missingness differs between scores, so each between-score comparison uses the intersection of available cases, with both AUROCs, the denominator, and the number of events reported on that set. Differences and 95% confidence intervals were calculated using DeLong covariance estimates. The original five comparisons against MELD, chosen as an established reference score, were adjusted by Holm’s method. At revision, all 15 pairwise comparisons were additionally examined, with a separate Holm adjustment across the 15 tests for each endpoint (Table A1, Table A2 and Table A3). These expanded analyses were not part of the original comparison set. Confidence intervals for differences are unadjusted 95% intervals; multiplicity-adjusted *p*-values govern the corresponding tests. No formal test comparing a score’s AUROC between the two component outcomes was performed. Differences between component estimates are descriptive, and significance for one component but not the other is not itself evidence of heterogeneity.

For a variable added to an existing model, the DeLong test is not the appropriate tool [21]. The incremental contribution of each endoscopic variable was therefore tested by likelihood-ratio comparison of nested logistic models, with ΔAUROC reported separately as a measure of improvement in ranking. The model was limited to Child–Pugh score, active bleeding at endoscopy and portal vein thrombosis, specified before model fitting on clinical grounds and earlier studies [16,17,18], rather than selected by univariable *p* values. ACLF was not added because its components and timing could not be independently verified, it overlaps conceptually with hepatic-severity assessment, and an additional term would increase model complexity with only 28 complete-case events. The events-per-variable ratio is reported as a descriptive measure, not a guarantee of adequate sample size [22]. Optimism was estimated by bootstrap with 1000 replicates, the model being refitted within each replicate, and calibration is reported as the optimism-corrected slope. Clinical utility was assessed by decision curve analysis.

Missing covariate data were handled in two ways. Complete-case analysis is presented first because it does not require an imputation model, while still being vulnerable to selection associated with missingness, with the analysed denominator given for every estimate. Multiple imputation by chained equations was then performed, with 25 imputations, an imputation model containing the outcome together with 23 variables, namely the three variables of the multivariable model, red wale markings, the five other admission scores and 14 clinical and laboratory measurements, continuous variables imputed by Bayesian ridge regression with posterior sampling, and pooling by Rubin’s rules. Binary variables were restored to 0 or 1 by thresholding the imputed value at 0.5; because thresholding a continuous imputation can bias a binary variable, the whole procedure was repeated with a logistic imputation model for the binary variables. Of the three binary variables, only red wale markings had any missing values (10 of 303). The sensitivity analysis used chained equations in which each variable was imputed by a model matched to its type: logistic regression with Bernoulli draws for binary variables and Bayesian ridge regression with draws from the predictive distribution for continuous ones, cycling through the variables for ten iterations in each of 25 imputations so that the imputed binary values also entered the imputation of the other variables; the two approaches are compared in Section 3.7.

The original analyses were performed in Python 3.12.3 (Python Software Foundation, Wilmington, DE, USA) using NumPy 2.4.4, pandas 3.0.2, SciPy 1.17.1, statsmodels 0.15.0, scikit-learn 1.8.0 and Matplotlib 3.10.8. The DeLong test, the bootstrap internal validation, the decision curve analysis, the type-matched imputation and the simulation of unrecorded events after transfer were implemented by the authors and are available from the corresponding author on reasonable request, together with the script that generates every table and figure in this article from the analytical dataset. All tests were two-sided, and *p* values below 0.05 were considered statistically significant. The additional revision analyses used Python 3.12.14, NumPy 2.3.5, pandas 2.2.3, SciPy 1.17.0 and scikit-learn 1.8.0. Additional AUROCs computed from positive/negative placement values were checked against the rank-based implementation in scikit-learn. Generative AI tools assisted language editing, manuscript revision and preparation; their use is disclosed in the Acknowledgments.

### 2.6. Ethics

The study was approved by the Ethics Committee of the Bucharest Emergency Clinical Hospital (approval no. 132/10.10.2020). Given the retrospective design and the exclusive use of pseudonymised records, the requirement for individual informed consent was waived, and the study was conducted in accordance with the Declaration of Helsinki.

## 3. Results

### 3.1. Cohort and Outcome

**Figure 1:**
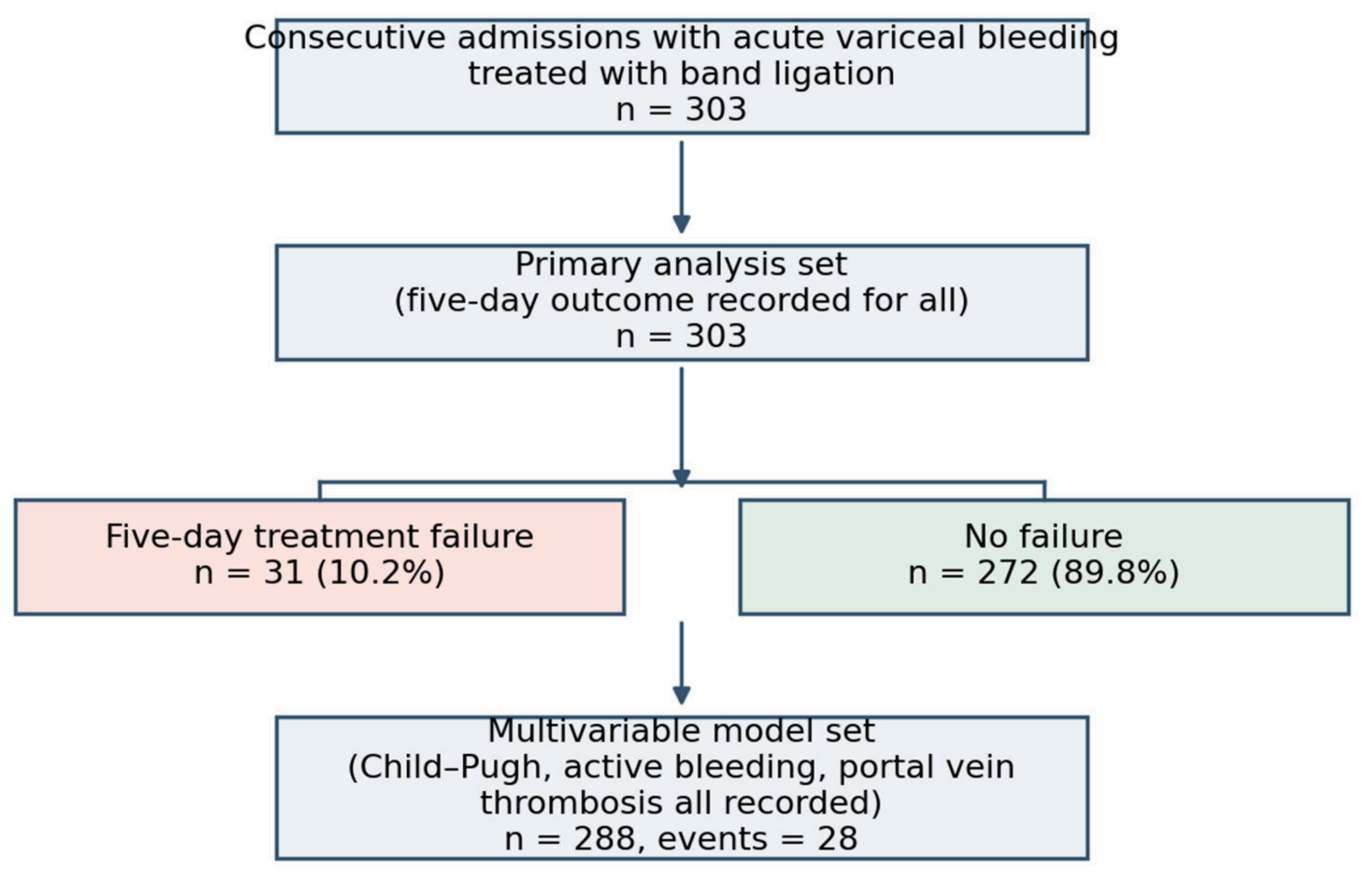
Flow of admissions and derivation of the analysis sets. A coded five-day outcome was available for every admission; this does not establish complete ascertainment after transfer.

The cohort comprised 303 consecutive admissions. Five-day treatment failure occurred in 31 (10.2%, 95% CI 7.3–14.2). A five-day outcome status was entered in the database for every admission, so none was excluded from the primary analysis (Figure 1); complete ascertainment, however, cannot be guaranteed for the 73 admissions transferred for shunting, in whom a rebleed at the receiving centre may not have been captured (Section 4.7). Failure comprised failure to control bleeding at the index endoscopy in 15 admissions and rebleeding within 120 h in 17, one admission contributing both.

Table 1 describes the cohort. Patients who subsequently failed had a higher Child–Pugh score and were more often found to be bleeding actively at endoscopy, while the MELD family of scores, the admission haemoglobin and the blood pressure did not differ appreciably between the two groups.

### 3.2. Care Delivered and Secondary Outcomes

A vasoactive drug was given in 279 of 303 admissions (92.1%), and antibiotic prophylaxis in the same proportion (Table 2). Endoscopy was performed within twelve hours in 240 of 303 admissions (79.2%), the interval to endoscopy being recorded for every admission. Technical haemostasis at the index procedure was achieved in 288 (95.0%). Sixty-three admissions (20.8%) were recorded as transferred for pre-emptive transjugular intrahepatic portosystemic shunting and ten (3.3%) for rescue shunting, the procedure being carried out at the receiving centre. Exact transfer and shunt-placement times were unavailable, so the portion of each 120-h window spent at the receiving centre and compliance with the recommended pre-emptive timing could not be verified. Urgent repeat banding was required in 34 (11.2%), balloon tamponade or a metal stent in 7 (2.3%), and post-banding ulcer bleeding was recorded in 13 (4.3%), of whom two had a ligation-site ulcer recorded as the source of rebleeding within 120 h. Among all 17 early rebleeds, the recorded source was oesophageal varices in eight, gastric varices in three, ligation-site ulcers in two and portal hypertensive gastropathy in one; the source was undocumented in three. Thus, the rebleeding endpoint is not confined to recurrent oesophageal variceal bleeding.

### 3.3. Discrimination of the Six Admission Scores

**Figure 2:**
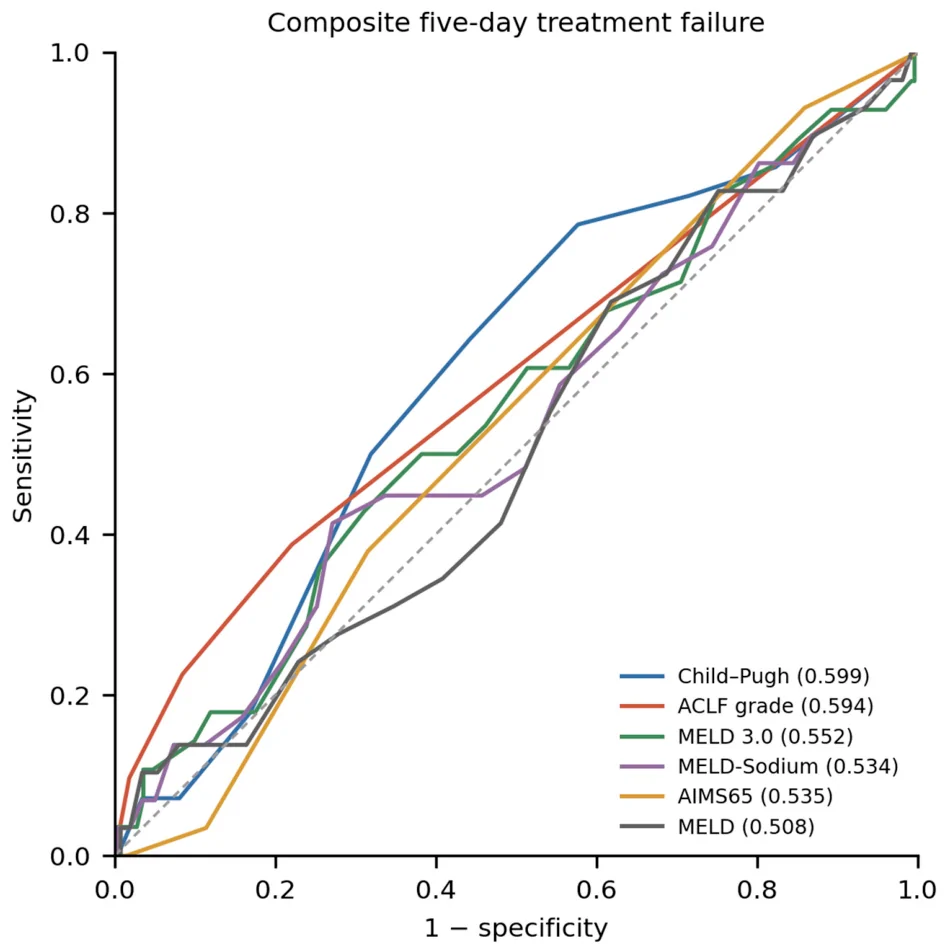
Receiver operating characteristic curves of the six admission scores for five-day treatment failure, each computed on its own complete cases. The dashed diagonal line indicates chance discrimination (AUROC = 0.5).

**Figure 3:**
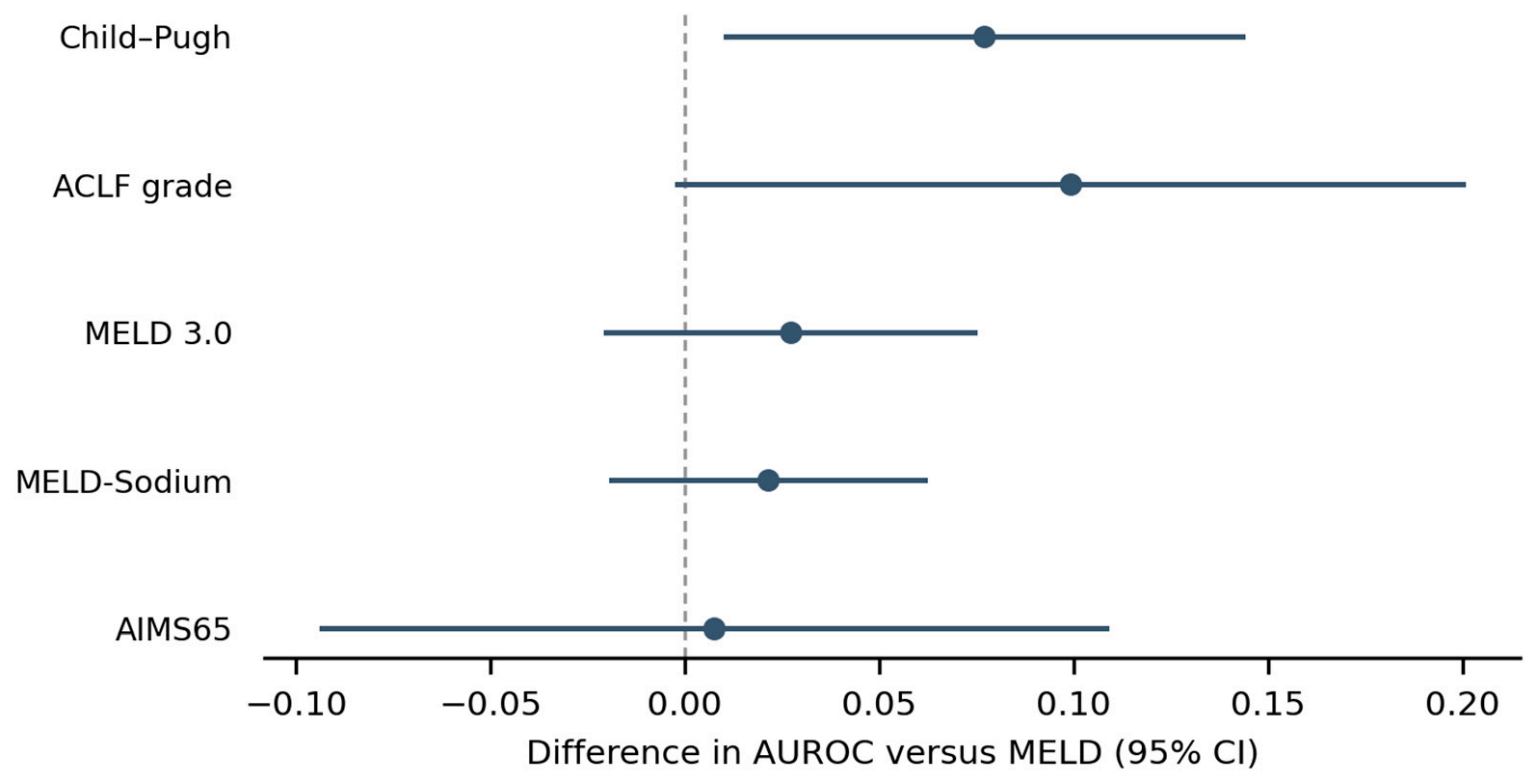
Paired differences in AUROC against MELD, with DeLong confidence intervals. Only the Child–Pugh interval excludes zero before multiplicity adjustment; no comparison is significant after the five-test Holm adjustment. The dashed vertical line marks no difference from MELD (difference in AUROC = 0).

All six scores discriminated poorly against the composite (Table 3, Figure 2). The areas under the curve ranged from 0.508 for MELD, an interval that includes chance, to 0.599 for the Child–Pugh score. Against MELD, the largest paired difference was +0.099 for the ACLF grade and +0.077 for Child–Pugh (Figure 3); the unadjusted *p*-values were 0.055 and 0.023, respectively, but no comparison survived Holm adjustment for the five tests, the smallest adjusted value being 0.116. The expanded set of all 15 paired comparisons also yielded no significant difference after Holm adjustment; the smallest adjusted *p*-value was 0.348 (Table A1). Clinically relevant differences remain compatible with the confidence intervals.

Using the recomputed scores and recorded ACLF grade, these data do not establish the superiority of one score for the composite endpoint. The recorded-score sensitivity analyses require separate interpretation (Section 3.8). With 31 events, the confidence intervals on the paired differences span roughly 0.1 AUROC units in each direction, so a difference of clinical size remains entirely compatible with them. The nominal ordering places the two measures of hepatic reserve and organ failure above most of the MELD family, but it is not identical across endpoints, since for rebleeding MELD 3.0 ranked above the ACLF grade, and it is not supported by a formal test.

### 3.4. Red Wale Markings and Five-Day Failure

Red wale markings were documented in 293 of the 303 admissions and were present in 159 of these (54.3%). They showed no detectable association with five-day treatment failure in this cohort. Failure occurred in 14 of the 159 admissions in which the sign was present (8.8%) and in 15 of the 134 in which it was absent (11.2%), an odds ratio of 0.77 (Fisher *p* = 0.558). Sensitivity was 48.3% (31.4–65.6) and specificity 45.1% (39.2–51.1), giving likelihood ratios of 0.88 for a positive and 1.15 for a negative finding. Both lie close enough to unity that the sign would be unlikely to shift a pre-test probability appreciably in either direction.

The result did not change on adjustment. In the 278 admissions with all four variables recorded, adding red wale markings to a model containing the Child–Pugh score, active bleeding at endoscopy and portal vein thrombosis did not improve fit (χ^2^ = 0.33, *p* = 0.563), and the adjusted odds ratio was 0.78 (95% CI 0.34–1.79, *p* = 0.563). The absence of the sign appeared no more informative than its presence in these data.

### 3.5. The Two Components of the Endpoint

**Figure 4:**
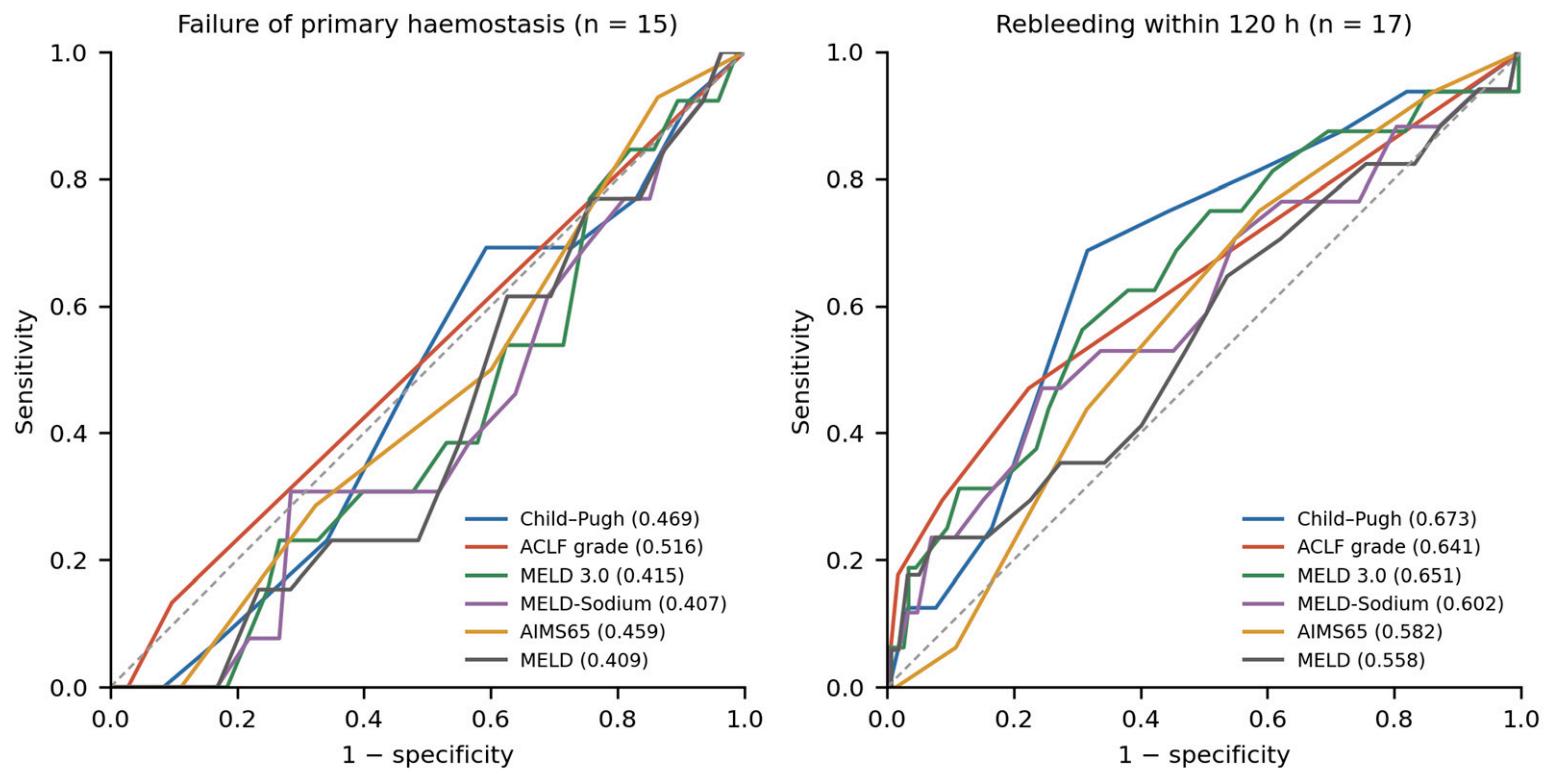
Receiver operating characteristic curves of the six admission scores for each component of the endpoint. The point estimate of discrimination was higher for rebleeding than for failure of primary haemostasis for every score, although the confidence intervals are wide and overlap. No formal between-component test was performed. Panel titles show whole-cohort event totals; score-specific analysed denominators and event counts are reported in Table 4. The dashed diagonal line indicates chance discrimination (AUROC = 0.5).

The composite endpoint comprises two events that plausibly differ in mechanism, and the point estimates of the scores differed between them (Table 4, Figure 4). The point estimate of discrimination for rebleeding within 120 h exceeded that for failure of primary haemostasis for all six scores, the gap ranging from 0.123 for AIMS65 to 0.236 for MELD 3.0 and 0.204 for Child–Pugh; for failure of primary haemostasis, the point estimates of five of the six scores lay below 0.5, the exception being the ACLF grade at 0.516. The confidence intervals are wide, as 15 and 17 events allow, and for most scores they overlap between the two components; no formal test of the difference between the two associations was performed, and the consistency of the direction across all six scores is the observation rather than any single contrast. Because the six scores are correlated with one another and were evaluated in the same patients, this consistency is not equivalent to six independent observations, and it does not establish that score performance differs between the two components.

The point estimates for active bleeding and portal vein thrombosis were larger for failure of primary haemostasis than for rebleeding. For active bleeding, the odds ratios were 4.52 (95% CI 1.40–14.53; Fisher *p* = 0.012) and 1.07 (0.40–2.90; *p* = 1.00), respectively. For portal vein thrombosis, they were 3.01 (0.98–9.26; *p* = 0.060) and 1.79 (0.56–5.75; *p* = 0.304). Red wale markings showed no clear association with either component: 0.71 (0.23–2.17) and 0.94 (0.35–2.52). These are unadjusted component associations with wide Wald confidence intervals; differences between the odds ratios were not formally tested. Active bleeding and technical haemostasis were assessed during the same procedure, creating potential temporal and definitional overlap (Section 4.4).

No comparison among the six primary score definitions reached significance for either component after Holm adjustment, either in the five comparisons with MELD or in the expanded 15-comparison sets (Table A2 and Table A3). In the latter sets, the minimum adjusted *p* values were 1.000 for primary haemostasis failure and 0.825 for rebleeding. Against rebleeding, the largest difference from MELD was +0.088 for Child–Pugh (95% CI −0.002 to +0.177, unadjusted *p* = 0.055, adjusted *p* = 0.275) and +0.081 for the ACLF grade. The component analysis is therefore compatible with a separation between the two kinds of predictors, but it neither establishes that separation nor ranks the scores against one another.

### 3.6. Endoscopic and Imaging Findings That Were Associated with Failure

**Figure 5:**
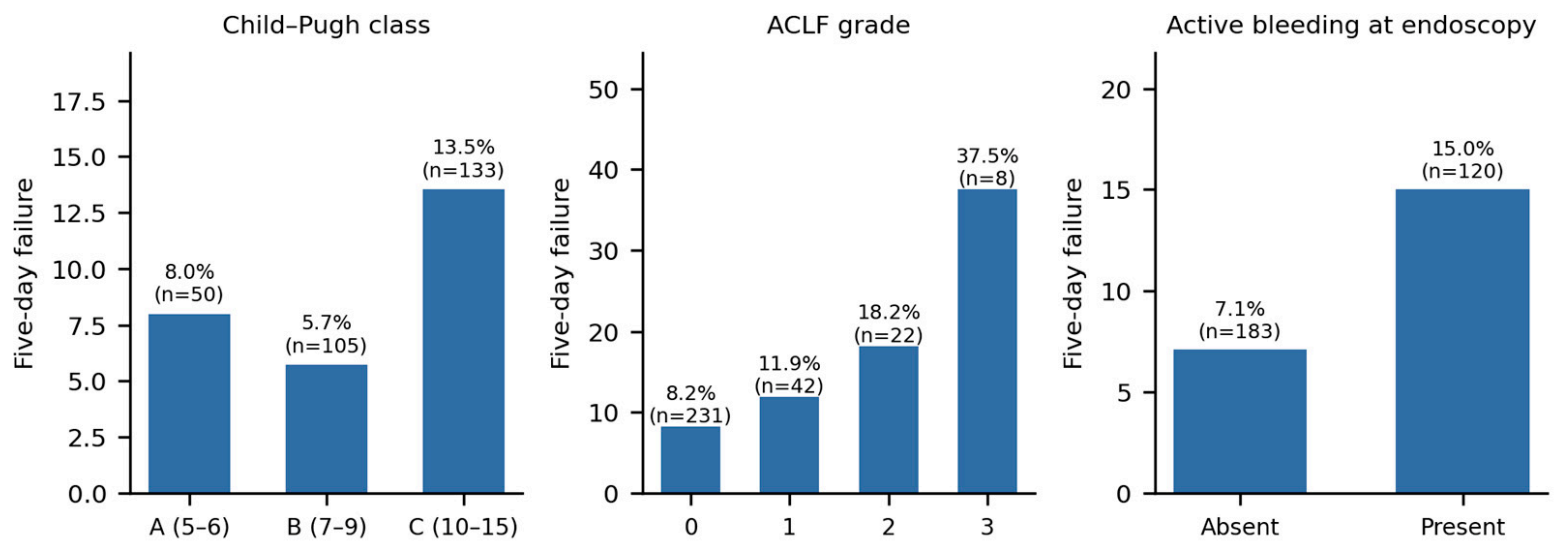
Five-day treatment failure by Child–Pugh class, by ACLF grade and by the presence of active bleeding at the index endoscopy.

Two findings available at the end of the index endoscopy were associated with the outcome (univariable and adjusted estimates are given in Section 3.7). Failure occurred in 18 of the 120 admissions with active bleeding at endoscopy (15.0%) against 13 of 183 without (7.1%), an unadjusted odds ratio of 2.31 (95% CI 1.09–4.91, *p* = 0.030). Portal vein thrombosis was present in 46 admissions, of which 9 failed (19.6%) against 22 of 257 without (8.6%), an odds ratio of 2.60 (1.11–6.08, *p* = 0.028). Failure also rose monotonically with the ACLF grade, from 8.2% at grade 0 to 37.5% at grade 3, and was highest in Child–Pugh class C, although the gradient across the three Child–Pugh classes was not monotonic (Figure 5). Table 5 gives the performance of conventional thresholds and of individual findings. These figures are descriptive: most likelihood ratios lie close to 1, and the high negative predictive values largely reflect the low prevalence of the endpoint (10.2%) rather than useful rule-out performance [23].

### 3.7. Multivariable Model and Internal Validation

**Figure 6:**
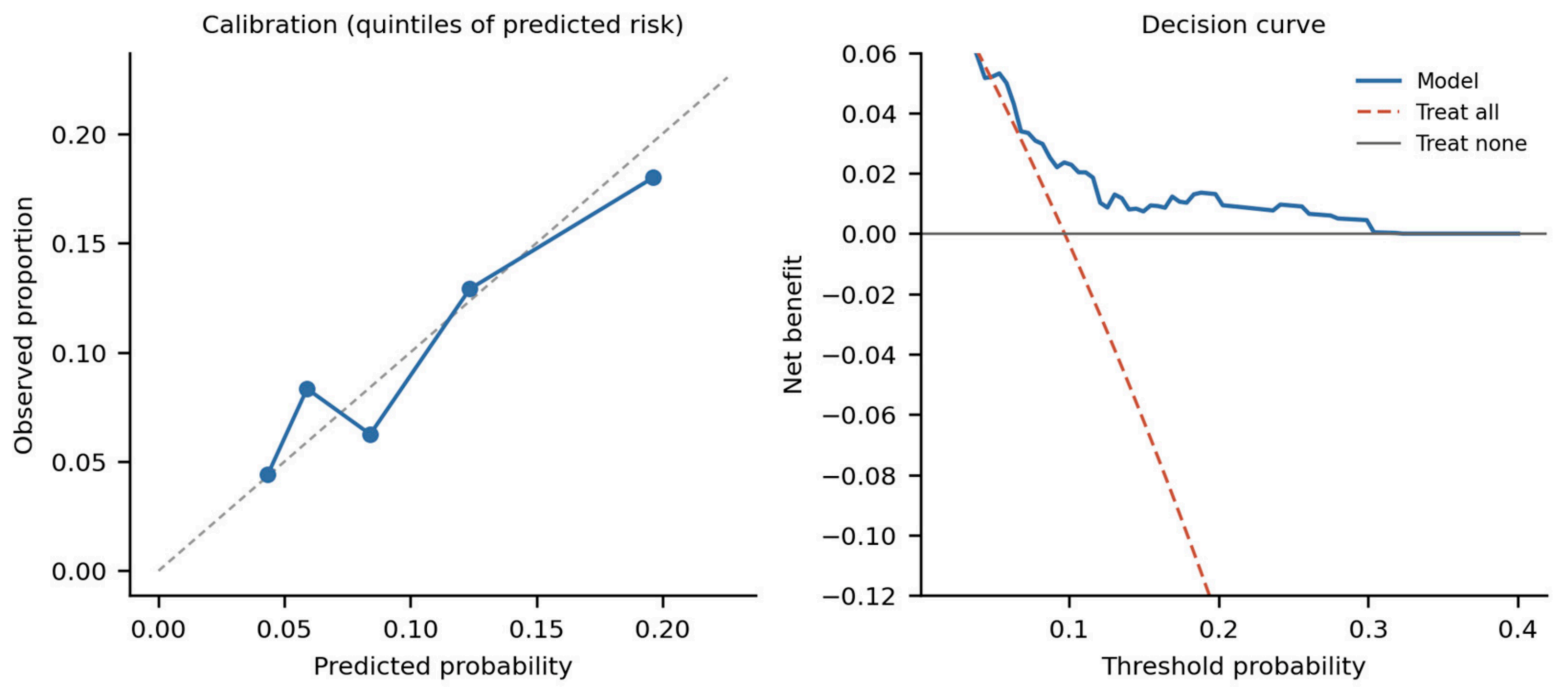
Calibration of the three-variable model across quintiles of predicted risk and decision curve analysis against the strategies of treating all and treating none. In the calibration panel, the dashed diagonal line indicates perfect agreement between predicted and observed risk; in the decision curve panel, the dashed line represents the treat-all strategy and the solid grey line the treat-none strategy.

A model containing the Child–Pugh score, active bleeding at endoscopy and portal vein thrombosis was fitted in the 288 admissions with all three variables recorded, of which 28 met the endpoint, a ratio of 9.3 events per variable. The three variables were specified before the analysis, on the basis of the earlier literature in which Child–Pugh class, active bleeding at endoscopy and portal vein thrombosis recur as independent predictors of five-day failure [16,17,18], and were not selected after inspection of the univariable results. The ACLF grade was not added, despite its univariable association (Table 6), for three reasons: it was the only score taken as recorded rather than recomputed; it overlaps with the Child–Pugh score as a measure of the severity of liver disease; and a fourth variable would have lowered the ratio to 7 events per variable, increasing the risk of unstable estimates [22,24]. Its apparent area under the curve was 0.660, and the optimism-corrected value was 0.628, with a corrected calibration slope of 0.878 (Figure 6). An optimism-corrected area under the curve of 0.628 indicates limited discrimination, and the model is regarded as exploratory rather than as a tool for clinical use. In the adjusted model the odds ratio was 1.11 per Child–Pugh point (95% CI 0.94–1.31, *p* = 0.231), 2.32 for active bleeding at endoscopy (1.03–5.21, *p* = 0.041) and 2.64 for portal vein thrombosis (1.05–6.61, *p* = 0.039).

Multiple imputation did not alter the direction of any estimate. The pooled odds ratio for active bleeding at endoscopy was 2.31 (1.06–5.01, *p* = 0.034) and for portal vein thrombosis 2.62 (1.09–6.28, *p* = 0.032), while the Child–Pugh score remained non-significant at 1.10 (0.94–1.29). After imputation, the AUROC of each of the six scores changed by less than 0.02 (Table 7). Repeating the imputation with type-matched chained equations, in which the binary variables were imputed by logistic regression, gave slightly different estimates: 2.34 (1.08–5.07) for active bleeding, 2.64 (1.10–6.33) for portal vein thrombosis and 1.08 (0.92–1.28, *p* = 0.340) for the Child–Pugh score, whose imputed area under the curve was 0.584 against 0.599. The direction and the approximate size of every estimate were preserved, but the *p*-value for the Child–Pugh term moved from 0.221 to 0.340, so the choice of imputation model is not entirely immaterial even though only one binary variable had missing values. Adding death within 120 h to the composite, which adds a single event, changed the discrimination of every score by less than 0.01 AUROC units. A further analysis addressed the 73 admissions transferred for shunting, in whom a rebleed occurring at the receiving centre might not have reached our records; failure of primary haemostasis, which occurred during the index procedure at the study centre, was not exposed to this risk. Ten of the 73 transferred admissions and 21 of the remaining 230 were recorded as having failed (13.7% and 9.1%). Because the transferred patients differed from the others in risk and in treatment, this comparison is descriptive only and says nothing about the completeness of ascertainment in either group. We then assumed that k of the 63 transferred admissions currently counted as non-events had had an unrecorded rebleed, allocated those events at random, and repeated the analysis in 2000 simulations for each value of k from 3 to 15, summarising each result as the mean and the 2.5th and 97.5th percentiles of the simulated values; these percentiles describe the variability of the simulation under the stated assumption and are not confidence intervals. With six unrecorded rebleeds, the area under the curve of the Child–Pugh score was 0.686 (simulation range 0.646–0.723) for rebleeding and 0.621 (0.594–0.647) for the composite, while failure of primary haemostasis was unchanged by construction. Across k = 3 to 15, the mean difference between the two components remained positive for all six scores, ranging from 0.074 to 0.268. The odds ratio for active bleeding at endoscopy was similar or slightly higher, 2.40 (1.66–3.31) at k = 6, whereas that for portal vein thrombosis fell to 2.37 (1.88–3.40) at k = 6 and 2.09 (1.36–3.16) at k = 15. If rebleeds had been missed in this way, the observed association with portal vein thrombosis would therefore overstate the association that complete ascertainment would have shown. These results are conditional on random allocation of the missing events; other mechanisms of under-ascertainment, for instance missed events concentrated among patients with low scores, were not modelled and could lead to different conclusions.

### 3.8. Additional Sensitivity Analyses

The recorded-versus-recomputed comparisons used identical available cases for each score (Table A4). For Child–Pugh, 277 admissions were comparable: recorded and recomputed AUROCs were 0.517 and 0.507 for technical haemostasis failure, 0.659 and 0.660 for rebleeding, and 0.613 and 0.609 for the composite. For MELD-Sodium, the corresponding values in 281 admissions were 0.452 and 0.417, 0.601 and 0.586, and 0.536 and 0.528. The direction of the descriptive component contrast persisted, but no formal between-component test was performed. Direct recorded-minus-recomputed differences were small relative to their uncertainty.

Substituting recorded values in the five comparisons against MELD changed some conclusions about individual score comparisons (Table A5). Recorded Child–Pugh exceeded MELD for the composite by 0.102 (95% CI 0.037–0.166; Holm *p* = 0.010; *n* = 280, 26 events) and for rebleeding by 0.118 (0.040–0.195; Holm *p* = 0.014; *n* = 280, 15 events). Both score values and available-case intersections differ from the primary analysis, so these results do not establish robust superiority. They demonstrate sensitivity to recording and case availability and are not used to select a preferred score.

Excluding the single admission with both components left 302 admissions, 14 technical failures, 16 rebleeds and 30 composite failures. The rebleeding AUROC remained numerically higher than the technical-failure AUROC for all six scores; for Child–Pugh the estimates were 0.702 and 0.489. Excluding the two recorded ligation-ulcer events from the rebleeding definition, while retaining those admissions as non-events for this restricted endpoint, left 15 rebleeds; the Child–Pugh AUROC was 0.659, and the numerical direction across components persisted for all six scores (Table A7). These checks address the influence of these particular records and definitions; they do not validate the event classification or resolve missing follow-up.

## 4. Discussion

In 303 consecutive admissions treated with band ligation, five-day treatment failure occurred in 10.2%. Against the composite endpoint, none of the six admission scores discriminated usefully, and no difference between them was established after Holm adjustment of either the original five comparisons with MELD or all 15 score pairs; this is an absence of evidence of a difference rather than evidence that the scores perform equivalently [25]. These statements refer to the primary score definitions; the recorded-score sensitivity analysis showed that some comparative *p*-values changed (Section 3.8). Separating the composite into its two components offers one possible explanation. Point estimates of discrimination were higher for rebleeding than for failure of primary haemostasis for each of the six scores, while those for the endoscopic findings tended in the opposite direction. With 15 and 17 events, respectively, this is an exploratory observation rather than a demonstration, and the most that can be said is that the direction was consistent across the six scores, without a formal test of the difference between the two components.

### 4.1. Why the Scores Perform Poorly

Areas under the curve between 0.51 and 0.60 for the composite are lower than most published series reports. Buckholz et al. obtained values near 0.77 for six-week mortality [9] and Kırdar et al. 0.888 for MELD against six-week mortality [10]. Part of the discrepancy is one of endpoints: those studies predicted death over six weeks, an outcome dominated by hepatic reserve, which is what MELD and Child–Pugh measure. A second possible part, which these data can only suggest, is the composition of the endpoint itself. The technical component used here may depend partly on procedural factors, which are not represented in biochemical scores; its restricted definition may itself contribute to the observed pattern. For this component the point estimate for MELD was 0.409. For rebleeding, the scores reached between 0.558 and 0.673, the three highest being Child–Pugh, MELD 3.0 and the ACLF grade at 0.641 to 0.673, which is broadly in keeping with what the literature reports for bleeding endpoints.

This reading is consistent with direct measurement in another cohort. Abraldes et al. measured the hepatic venous pressure gradient within 48 h in 117 patients and found that a gradient of 20 mmHg or more, a systolic pressure below 100 mmHg and a non-alcoholic aetiology predicted five-day failure, the clinical model reaching a c statistic of 0.79 [26]. That suggests the portal pressure itself carries much of the signal, and none of the six scores measures it. Mortality-prediction studies address a different endpoint. Bai et al. validated the existing CAGIB score in 2467 patients and separately developed machine-learning models from its components [27]. Tsai et al. reported an AUROC of 0.866 for in-hospital mortality across 32,826 emergency department visits [28]. The new machine-learning models still require external validation, and these mortality results do not establish performance for early bleeding failure.

### 4.2. Treatment as a Competing Explanation

The composition of the endpoint is not the only explanation available, and a second one has to be set beside it. Of the 146 admissions recorded as eligible for pre-emptive shunting, 63, a fifth of the whole cohort, were transferred and received it, and a further ten were transferred for rescue shunting. Pre-emptive shunting is offered to patients judged to be at high risk, a judgement in which the admission scores take part, and it reduces the risk of rebleeding. Treatment may therefore act against the association it is being used to measure: the patients with the worst scores may be the ones most likely to have received the intervention that prevents the outcome. In a cohort in which a fifth of patients are treated in this way, the attenuation might be appreciable, and it cannot be separated from the effect of the endpoint structure with these data. A design able to distinguish them would need the reasons for which 83 of the 146 patients recorded as eligible did not receive preemptive TIPS, the time at which the decision was made, and a comparison free of confounding by indication; neither the reasons nor the timing were recorded here.

Pre-emptive TIPS is an intervening treatment for the rebleeding outcome, in addition to being associated with transfer and potentially incomplete follow-up. Its ability to reduce failure or rebleeding is supported by the early-TIPS trial [29]. In our data, the 63 admissions recorded as receiving preemptive TIPS had higher median Child–Pugh and MELD scores than the other 240 admissions (11 versus 9 and 21 versus 16, respectively), while recorded rebleeding occurred in 2/63 (3.2%) versus 15/240 (6.3%) (Table A6). This descriptive contrast is compatible with treatment affecting the relation between baseline risk and subsequent events, but confounding by indication, uncertain treatment classification and differential ascertainment prevent a causal interpretation. TIPS does not preclude all later bleeding. Treating it as a competing event would target bleeding before TIPS, whereas simple censoring at TIPS could be informative because treatment depends on risk. A hypothetical no-TIPS analysis would require explicitly defined assumptions, treatment times and sufficient information on treatment selection [30]. These requirements are not met here. Restricting the analysis to the 230 non-transferred admissions would not remove selection bias; that subgroup contains 21 composite failures but only 13 recorded rebleeds. We therefore present observed outcomes under the local treatment pathway and do not estimate untreated risk or TIPS effectiveness.

### 4.3. Red Wale Markings

Red wale markings entered the prognostic literature through the North Italian Endoscopic Club index, where they predicted a first variceal haemorrhage [14]. The present study concerns prognosis after an index bleed treated with ligation. In this setting, we did not demonstrate a clear association with five-day failure. The point estimates of the likelihood ratios, 0.88 and 1.15, suggest little discrimination in this sample, but the uncertainty around the association precludes concluding that the sign has no prognostic information.

Red wale markings are recorded routinely and guide decisions about primary prophylaxis, and nothing here bears on that use. What these data do not provide is evidence that the sign carries prognostic weight once an index bleed has been treated; with an adjusted odds ratio of 0.78 and an interval running from 0.34 to 1.79, they remain compatible with clinically relevant effects in either direction, and they do not support a recommendation about how the sign should or should not be used after ligation.

### 4.4. What Did Predict Failure

Active bleeding at the index endoscopy was associated with approximately twice the odds of composite failure. Its component odds ratios were 4.52 for technical haemostasis failure and 1.07 for rebleeding, but their difference was not formally tested and both estimates are imprecise. The finding is compatible with technical difficulty during active bleeding; it does not demonstrate higher portal pressure or a distinct causal mechanism. The predictor and technical outcome were recorded during the same procedure, so temporal proximity, shared documentation and classification practices may contribute to the association. Four of the 15 admissions with technical failure were coded without active bleeding, indicating that these fields are not interchangeable. The records available for analysis do not explain whether this reflects timing within the procedure, technical difficulty or classification differences. Portal vein thrombosis had point estimates in the same direction, but these likewise do not establish different determinants between components.

These two associations recur in the literature on this endpoint, and the older studies appear to have captured similar relationships. Ben-Ari et al., in 695 consecutive admissions, identified active bleeding at endoscopy as an independent predictor of failure to control bleeding [16]. D’Amico and De Franchis reported active bleeding at endoscopy, haematocrit, aminotransferase, Child–Pugh class and portal vein thrombosis as the significant predictors of five-day failure [17]. Amitrano et al., in 185 unselected cirrhotic patients found Child–Pugh class C, a white cell count above 10 × 10^9^/L and portal vein thrombosis to be the only independent predictors [18]. Those lists combine clinical, endoscopic and imaging variables but do not assign them definitively to separate components. Guo et al. additionally identified a shock index above 1.2 [31], whereas in our cohort the shock index, recomputed for every admission, did not differ between those who failed and those who did not (median 1.20 against 1.10, *p* = 0.24).

### 4.5. Comparison with the Organ-Failure Literature

The marginal AUROC point estimate for ACLF grade was numerically close to that for Child–Pugh and showed a monotonic gradient, from 8.2% failure at grade 0 to 37.5% at grade 3. Zhu and Jiang, in an intensive-care database, found acute-on-chronic liver failure in 54.0% of their cohort and reported the CLIF-C scores as the best predictors [12], and Kurup et al. assessed organ failure at admission and again at 48 h in a multicentre Australian cohort [13]. Our cohort is a ward population with a much lower prevalence of organ failure, which limits the comparison; the grade 3 subgroup contained only eight admissions, so the apparent gradient rests on very few events at its upper end. The ACLF grade was also the only score taken as recorded rather than recomputed (Section 2.3), so its accuracy and timing could not be verified in the same way, and its nominal position close to the Child–Pugh score should be interpreted with this asymmetry in mind.

### 4.6. Clinical Implications

Two statements are supported and a third is not. These data do not identify any admission score as preferable to another for this endpoint: the nominal ordering places the Child–Pugh score and the ACLF grade above the MELD family, but no difference using the primary score definitions survives adjustment for either five or 15 comparisons, and a claim that one score should replace another would go beyond what 31 events can support. What the data may suggest, with the reservations given in Section 4.4, is that part of the information relevant to the procedural component is already in the endoscopy report and the admission imaging, and that a score computed from the admission laboratory values may relate more to the later component than to the earlier one.

Eligibility for preemptive shunting by the recorded criteria was noted in 146 of the 285 admissions in which it was documented, and 63 of these, a fifth of the whole cohort, were transferred and received the procedure; the remaining 83 eligible patients did not receive preemptive TIPS, three of whom were later transferred for rescue shunting, and the records do not state why the preemptive procedure was not performed, and eligibility was not documented in 18 admissions. Recorded eligibility, the final clinical indication and the treatment actually given are therefore not interchangeable here. Because the procedure is not available at the study centre, the practical decision that a prognostic tool might inform here is whether and when to transfer, and it has to be taken on the information available at the end of the index endoscopy. The criteria remain those of Baveno VII, supported by the individual patient data meta-analysis of Nicoară-Farcău et al. [32] and by the work of Lv et al. on concordance for benefit in Child–Pugh B cirrhosis [33]. Nothing in these data bears on that decision, which is a question of treatment benefit rather than of prognostic discrimination, and our results should not be used to modify it.

### 4.7. Limitations

This is a single-centre retrospective study with four principal limitations: statistical power, the definition and ascertainment of the endpoint, possible under-ascertainment of rebleeding after transfer, and the intervening effect of pre-emptive shunting. The first is statistical power. With 31 events in the composite and 15 and 17 in its components, every confidence interval is wide, and the negative findings are failures to demonstrate an association rather than demonstrations of its absence. No comparison using the primary score definitions survived multiplicity adjustment, so that ordering is nominal. Recorded-score sensitivity analyses changed some comparisons with MELD, underlining their dependence on score recording and case availability. The contrast between the two components rests on the consistency of a direction across six scores rather than on any single significant test, and it is presented as a hypothesis for prospective study.

Second, the endpoint was not adjudicated against written criteria. The criteria were those of routine departmental practice and could not be reconstructed from the files; the sources used to classify each component are described in Section 2.4, but classification was not blinded to the admission variables. Because the central question depends on distinguishing the two components accurately, misclassification between them, for instance, of an early rebleed as failure of primary haemostasis or the reverse, could either create or obscure the pattern reported here. The technical-failure definition is narrower than full clinical failure-to-control criteria, and the rebleeding component includes several bleeding sources. A prospective study should adjudicate both components using explicit consensus-compatible criteria, document initial control and recurrent bleeding, and apply a consistent time origin from the initial hospital presentation [4]. Our centre-based time origin may miss events before referral and include events beyond five days from the initial presentation.

Third, and potentially more consequential, ascertainment may have differed between the two components. Seventy-three admissions were transferred for shunting; a five-day outcome is recorded for all of them, but the records do not document how an event occurring after transfer was ascertained at the receiving centre. Failure of primary haemostasis necessarily occurred at the study centre and was not exposed to this risk, whereas a rebleed at the receiving centre may have been missed. The simulation in Section 3.7 explores one mechanism, random allocation of unrecorded rebleeds, under which the contrast between the components persisted; it cannot exclude other mechanisms, for instance missed events concentrated among patients with low or high scores, which could strengthen, weaken or reverse the contrast.

Fourth, preemptive shunting is not only a source of missing follow-up but also an intervening treatment that lowers the risk of rebleeding and is offered to patients selected for higher risk (Section 4.2). The rebleeding component therefore reflects subsequent management as well as admission risk, and the two cannot be separated with these data.

Eligibility requires band ligation, so patients who died before endoscopy or in whom ligation was not feasible are absent by construction. Red wale markings and active bleeding were abstracted from routine reports without standardised image re-review or assessment of inter-observer agreement. Computer-assisted endoscopy may help standardise assessment of high-risk variceal features, but this is a potential application rather than a validated remedy for the measurement limitations of this cohort; prospective external validation remains necessary [34]. Endoscopy was performed within twelve hours in 79.2% of admissions. Following resuscitation, guidelines recommend endoscopy within twelve hours, or as soon as safely possible, in unstable patients [4,5,6]. Possible contributors to delay in this emergency setting include competing emergencies, availability of staff and equipment, stabilisation and timing of initial assessment; these reasons were not systematically quantified, so individual delays cannot be attributed to them. Observational cohorts reported corresponding proportions of 63.1% [35] and 14.0% [36], but such comparisons do not establish that delay was harmless here. The quality of the score components is another limitation. Recalculation standardises arithmetic but cannot verify clinical grading, dialysis status, assessment timing or the underlying source values. The recorded-versus-recomputed analyses are now reported in Section 3.8 and Table A4 and Table A5; they show that some inferences about comparative performance depend on recording and case availability. ACLF classification and timing could not be independently validated, creating a possibility that it incorporated information acquired after the intended baseline. The AIMS65 mental status criterion was approximated by encephalopathy grade. The hepatic venous pressure gradient was not measured, and no causal mechanism can be tested directly.

Verified longer-term vital status was unavailable, so no inference about post-discharge survival is made. Sequential admission identifiers also prevent direct identification of repeat admissions. Prior variceal bleeding was recorded in 89 of 303 admissions. Only under the unverified assumption that every repeat admission was consistently recorded as having prior bleeding would this imply at least 214 distinct patients. This conditional bound does not identify patient clusters, establish the effective sample size or quantify the effect on uncertainty. Repeated observations may make independence-based confidence intervals too narrow and *p*-values too small; patient-level resampling or cluster adjustment was not possible. These limitations are additional reasons to regard the estimates as exploratory.

### 4.8. Contrast with the Prior Literature on Composite Predictors

Published studies have evaluated scores against different endpoints, including composite bleeding failure and mortality. These endpoints should not be treated as interchangeable when interpreting performance. Buckholz et al., in a multicentre US cohort of 354 patients, compared MELD, MELD 3.0 and Child–Pugh and reported similar discrimination for six-week mortality without decomposing the outcome into its early components [9]. Kırdar et al. found the Child–Pugh and MELD scores more accurate than newer non-invasive fibrosis indices, again against an undivided prognostic endpoint [10]. Demiröz et al. compared clinical and inflammatory scores for mortality and for rebleeding as two separately modelled outcomes rather than as components of one shared five-day endpoint [11]. Among the older studies addressing five-day failure directly, Ben-Ari et al., in 695 consecutive admissions, identified active bleeding at endoscopy as an independent predictor of failure to control bleeding, without separating that event from later rebleeding [16]. D’Amico and De Franchis, in a large prospective series, reported active bleeding, haematocrit, aminotransferase, Child–Pugh class and portal vein thrombosis together as predictors of the composite five-day failure [17]. Amitrano et al., in 185 unselected cirrhotic patients found Child–Pugh class C, the white cell count and portal vein thrombosis to be the only independent predictors of the same undivided endpoint [18]. These studies do not directly test whether the predictors have different associations with the two locally defined components examined here. The present study is, to our knowledge, among the few to examine this decomposition directly, and it offers one possible explanation for why such heterogeneous predictor lists recur across the literature: if the two events have different determinants, as these data suggest but cannot establish, a predictor list drawn from the composite would be expected to reflect a mixture of both.

The present contribution is a transparent description of component-specific estimates in a consecutive clinical cohort, not a recommendation to select one score over another. The expanded pairwise analyses and recorded-score checks improve the assessment of uncertainty but cannot repair non-standardised outcome classification, incomplete post-transfer ascertainment or treatment-related selection. Prospective studies should prespecify consensus-compatible component definitions, record patient identifiers and treatment times, verify outcomes across centres and test differences between components directly.

## 5. Conclusions

In this cohort of 303 consecutive admissions for acute variceal bleeding, six admission scores discriminated poorly for the composite five-day endpoint, and no between-score difference using the primary definitions was established after adjustment for multiplicity. Some comparisons changed when recorded scores were substituted, warranting caution about their stability. Separating the composite into its two components suggests one possible reason, without establishing it. Point estimates of discrimination were higher for rebleeding than for failure of primary haemostasis for each of the six scores, although the difference was not formally tested, while active bleeding was associated with technical haemostasis failure, and its association with rebleeding remained uncertain; red wale markings showed no clear association with either. These are exploratory findings resting on 15 and 17 events, and a competing explanation exists, since a fifth of the cohort was transferred for preemptive shunting, which may attenuate the association between an admission score and a later event, and rebleeding after transfer may have been under-ascertained. These observations support reporting the two components of the endpoint separately in studies of early failure, while documenting outcome definitions, ascertainment and intervening treatment. The observed pattern suggests that the components of five-day treatment failure may capture different clinical processes, a hypothesis requiring prospective confirmation in a larger cohort.

## Figures and Tables

**Table 1:** Baseline characteristics of the 303 admissions by five-day treatment failure. Continuous variables are median (interquartile range); categorical variables are *n* (%). Every percentage and every *p*-value is computed on the admissions in which the variable was recorded, the number missing being given in the last column. The Child–Pugh score and the class derived from it are the values recomputed from the five components, which are missing whenever any component is missing.

Variable	All (*n* = 303)	No Failure (*n* = 272)	Failure (*n* = 31)	*p*	Missing
Age, years	58.0 (50.0–66.0)	58.0 (50.8–67.0)	60.0 (49.0–64.5)	0.783	0
Child–Pugh score	9.0 (7.0–11.0)	9.0 (7.0–11.0)	10.5 (9.0–11.0)	0.084	15
MELD	17.0 (14.0–21.0)	17.0 (14.0–21.0)	17.0 (14.0–21.0)	0.890	12
MELD-Sodium	19.0 (14.0–24.0)	19.0 (14.0–23.8)	18.0 (15.0–24.0)	0.548	16
MELD 3.0	19.0 (15.0–24.0)	19.0 (14.5–24.0)	21.0 (15.0–25.0)	0.363	24
AIMS65	2.0 (1.0–3.0)	2.0 (1.0–3.0)	2.0 (1.0–3.0)	0.526	11
Haemoglobin, g/dL	7.9 (6.7–9.2)	8.1 (6.7–9.3)	7.3 (6.6–8.7)	0.138	0
Systolic blood pressure, mmHg	100.0 (90.0–114.0)	100.0 (90.0–114.2)	100.0 (90.5–110.0)	0.914	0
Heart rate, beats/min	110.0 (94.0–124.0)	110.0 (93.0–122.2)	118.0 (98.0–130.0)	0.052	0
INR	1.7 (1.4–2.0)	1.7 (1.4–1.9)	1.7 (1.5–2.0)	0.698	3
Bilirubin, mg/dL	2.4 (1.6–3.8)	2.4 (1.6–3.8)	2.7 (1.7–4.6)	0.431	4
Albumin, g/dL	3.0 (2.6–3.4)	3.0 (2.7–3.4)	2.9 (2.4–3.3)	0.322	8
Creatinine, mg/dL	1.1 (0.8–1.3)	1.1 (0.8–1.3)	1.1 (0.9–1.3)	0.503	5
Sodium, mmol/L	136.0 (133.0–139.0)	136.0 (133.0–139.0)	134.0 (132.5–138.0)	0.242	5
Platelets, ×10^9^/L	77.0 (53.0–109.8)	77.0 (53.0–109.0)	73.0 (53.0–124.0)	0.575	5
Lactate, mmol/L	2.1 (1.5–3.1)	2.1 (1.5–3.0)	2.2 (1.5–3.6)	0.707	28
Largest varix, mm	13.1 (10.5–16.3)	12.9 (10.4–15.9)	15.0 (11.9–17.0)	0.095	19
Red cell units in 24 h	2.0 (0.0–3.0)	2.0 (0.0–3.0)	3.0 (1.5–3.0)	0.060	0
Female sex	83 (27.4)	73 (26.8)	10 (32.3)	0.528	0
Alcohol aetiology	111 (36.6)	100 (36.8)	11 (35.5)	1.000	0
Child–Pugh class C	133 (46.2)	115 (44.2)	18 (64.3)	0.048	15
ACLF at admission	72 (23.8)	60 (22.1)	12 (38.7)	0.046	0
Hepatic encephalopathy ≥ grade 2	61 (20.1)	52 (19.1)	9 (29.0)	0.235	0
Infection at admission	71 (23.4)	62 (22.8)	9 (29.0)	0.502	0
Hepatocellular carcinoma	36 (11.9)	31 (11.4)	5 (16.1)	0.391	0
Portal vein thrombosis	46 (15.2)	37 (13.6)	9 (29.0)	0.033	0
Previous variceal bleeding	89 (29.4)	83 (30.5)	6 (19.4)	0.219	0
Haemodynamic instability	205 (67.7)	180 (66.2)	25 (80.6)	0.110	0
Active bleeding at endoscopy	120 (39.6)	102 (37.5)	18 (58.1)	0.033	0
Red wale markings present	159 (54.3)	145 (54.9)	14 (48.3)	0.558	10

**Table 2:** Process measures and outcomes. Percentages are given with Wilson confidence intervals; the interval to endoscopy was recorded for every admission.

Process Measure or Outcome	*n*/*N*	% (95% CI)
Vasoactive drug administered	279/303	92.1 (88.5–94.6)
Antibiotic prophylaxis	279/303	92.1 (88.5–94.6)
Endoscopy within 12 h	240/303	79.2 (74.3–83.4)
Technical haemostasis at index endoscopy	288/303	95.0 (92.0–97.0)
Transferred for pre-emptive TIPS	63/303	20.8 (16.6–25.7)
Transferred for rescue TIPS	10/303	3.3 (1.8–6.0)
Urgent repeat banding	34/303	11.2 (8.1–15.3)
Balloon tamponade or metal stent	7/303	2.3 (1.1–4.7)
Post-banding ulcer bleeding	13/303	4.3 (2.5–7.2)
Five-day treatment failure	31/303	10.2 (7.3–14.2)

**Table 3:** Discrimination for the composite five-day endpoint. Every comparison against MELD is computed on the intersection of the two scores; the denominator, the number of events, and both areas under the curve on that intersection are given so that each difference can be traced. Marginal areas under the curve with confidence intervals, computed on each score’s own complete cases, are given in Table 4.

Score	*n*	Events	AUROC on the Paired Set	AUROC of MELD in the Same Patients	Δ vs. MELD (95% CI)	*p*	Holm *p*
Child–Pugh	283	28	0.597	0.520	+0.077 (+0.011 to +0.143)	0.023	0.116
ACLF grade	291	29	0.607	0.508	+0.099 (−0.002 to +0.200)	0.055	0.219
MELD 3.0	279	28	0.552	0.525	+0.027 (−0.020 to +0.074)	0.261	0.784
MELD-Sodium	287	29	0.534	0.513	+0.021 (−0.019 to +0.062)	0.297	0.784
AIMS65	283	28	0.528	0.520	+0.007 (−0.093 to +0.108)	0.885	0.885
MELD	291	29	0.508 (0.397–0.618)	—	reference	—	—

**Table 4:** Discrimination of the six admission scores for each component of the endpoint and for the composite. Areas under the curve with 95% confidence intervals, computed on the admissions in which each score was available. Each cell also reports the analysed *n* and event count; these vary with score availability. The whole-cohort totals are 15, 17 and 31 events, respectively.

Score	Failure of Primary Haemostasis	Rebleeding Within 120 h	Composite
Child–Pugh	0.469 (0.320–0.617) *n* = 288; events = 13	0.673 (0.538–0.807) *n* = 288; events = 16	0.599 (0.491–0.707) *n* = 288; events = 28
ACLF grade	0.516 (0.396–0.636) *n* = 303; events = 15	0.641 (0.506–0.776) *n* = 303; events = 17	0.594 (0.498–0.689) *n* = 303; events = 31
MELD 3.0	0.415 (0.271–0.559) *n* = 279; events = 13	0.651 (0.502–0.800) *n* = 279; events = 16	0.552 (0.435–0.670) *n* = 279; events = 28
MELD-Sodium	0.407 (0.260–0.554) *n* = 287; events = 13	0.602 (0.446–0.759) *n* = 287; events = 17	0.534 (0.419–0.650) *n* = 287; events = 29
AIMS65	0.459 (0.323–0.594) *n* = 292; events = 14	0.582 (0.454–0.709) *n* = 292; events = 16	0.535 (0.436–0.634) *n* = 292; events = 29
MELD	0.409 (0.270–0.548) *n* = 291; events = 13	0.558 (0.404–0.712) *n* = 291; events = 17	0.508 (0.397–0.618) *n* = 291; events = 29

**Table 5:** Performance of score thresholds and of individual findings for five-day treatment failure. Each row is computed on the admissions in which that variable was recorded. PPV, positive predictive value; NPV, negative predictive value; LR, likelihood ratio. These analyses are descriptive; with an event prevalence of 10.2%, high negative predictive values are expected even for findings with little discriminating ability, and they should not be read as evidence of rule-out performance.

Threshold or Finding	Sensitivity, %	Specificity, %	PPV, %	NPV, %	LR+	LR−	*n*
MELD ≥ 20	31.0	65.3	9.0	89.5	0.89	1.06	291
MELD-Sodium ≥ 20	44.8	54.3	9.9	89.7	0.98	1.02	287
MELD 3.0 ≥ 20	53.6	53.8	11.5	91.2	1.16	0.86	279
Child–Pugh ≥ 10 (class C)	64.3	55.8	13.5	93.5	1.45	0.64	288
AIMS65 ≥ 2	65.5	41.1	10.9	91.5	1.11	0.84	292
ACLF grade ≥ 1	38.7	77.9	16.7	91.8	1.75	0.79	303
Red wale markings present	48.3	45.1	8.8	88.8	0.88	1.15	293
Active bleeding at endoscopy	58.1	62.5	15.0	92.9	1.55	0.67	303
Portal vein thrombosis	29.0	86.4	19.6	91.4	2.13	0.82	303

**Table 6:** Univariable and multivariable associations with five-day treatment failure. The adjusted column is the three-variable model; red wale markings are shown adjusted for the same three variables. OR, odds ratio. MELD and ACLF were not included in the prespecified three-variable model; “not included” does not denote exclusion by stepwise or univariable screening.

Variable	Unadjusted OR (95% CI)	*p*	Adjusted OR (95% CI)	*p*
Child–Pugh score, per point	1.14 (0.97–1.35)	0.110	1.11 (0.94–1.31)	0.231
Active bleeding at endoscopy	2.31 (1.09–4.91)	0.030	2.32 (1.03–5.21)	0.041
Portal vein thrombosis	2.60 (1.11–6.08)	0.028	2.64 (1.05–6.61)	0.039
Red wale markings present	0.77 (0.36–1.65)	0.496	0.78 (0.34–1.79)	0.563
MELD, per point	1.01 (0.94–1.09)	0.773	not included	–
ACLF grade, per grade	1.73 (1.16–2.58)	0.008	not included	–

**Table 7:** Complete-case and imputed discrimination of the six admission scores. Imputed values are the mean across 25 imputations.

Score	Complete-Case AUROC	Imputed AUROC	Difference
Child–Pugh	0.599	0.599	+0.001
ACLF grade	0.594	0.594	+0.000
MELD 3.0	0.552	0.552	−0.001
MELD-Sodium	0.534	0.545	+0.011
AIMS65	0.535	0.525	−0.010
MELD	0.508	0.526	+0.018

## Data Availability

The analysis code used for the results, tables and figures is available from the corresponding author on reasonable request. The anonymised analytical dataset is available from the corresponding author on reasonable request, subject to institutional approval.

## Supplementary Materials

The following supporting information can be downloaded at: https://www.mdpi.com/article/10.3390/diagnostics16213620/s1, Table S1: STROBE Statement—Checklist of items that should be included in reports of cohort studies; Table S2: TRIPOD Checklist: Prediction Model Development.

## Abbreviations

The following abbreviations are used in this manuscript:

ACLF	acute-on-chronic liver failure
AUROC	area under the receiver operating characteristic curve
CI	confidence interval
EVL	endoscopic variceal ligation
IQR	interquartile range
LR+/LR−	positive and negative likelihood ratio
MELD	model for end-stage liver disease
NPV	negative predictive value
OR	odds ratio
PPV	positive predictive value
TIPS	transjugular intrahepatic portosystemic shunt

## Appendix A. Additional Analyses

**Table A1:** All 15 paired comparisons for composite.

Comparison First vs. Second	*n*/Events	Paired AUROCs First/Second	Difference 95% CI	*p*	Holm *p*
Child–Pugh vs. ACLF grade	288/28	0.599/0.614	−0.015 (−0.119 to +0.088)	0.773	1.000
Child–Pugh vs. MELD 3.0	279/28	0.600/0.552	+0.048 (−0.014 to +0.110)	0.129	1.000
Child–Pugh vs. MELD-Sodium	279/28	0.600/0.545	+0.055 (−0.010 to +0.120)	0.096	1.000
Child–Pugh vs. AIMS65	288/28	0.599/0.531	+0.068 (−0.021 to +0.156)	0.133	1.000
Child–Pugh vs. MELD	283/28	0.597/0.520	+0.077 (+0.011 to +0.143)	0.023	0.348
ACLF grade vs. MELD 3.0	279/28	0.616/0.552	+0.063 (−0.039 to +0.165)	0.224	1.000
ACLF grade vs. MELD-Sodium	287/29	0.609/0.534	+0.075 (−0.025 to +0.174)	0.140	1.000
ACLF grade vs. AIMS65	292/29	0.607/0.535	+0.072 (−0.025 to +0.169)	0.143	1.000
ACLF grade vs. MELD	291/29	0.607/0.508	+0.099 (−0.002 to +0.200)	0.055	0.766
MELD 3.0 vs. MELD-Sodium	279/28	0.552/0.545	+0.007 (−0.024 to +0.039)	0.650	1.000
MELD 3.0 vs. AIMS65	279/28	0.552/0.532	+0.021 (−0.078 to +0.120)	0.680	1.000
MELD 3.0 vs. MELD	279/28	0.552/0.525	+0.027 (−0.020 to +0.074)	0.261	1.000
MELD-Sodium vs. AIMS65	279/28	0.545/0.532	+0.014 (−0.085 to +0.112)	0.788	1.000
MELD-Sodium vs. MELD	287/29	0.534/0.513	+0.021 (−0.019 to +0.062)	0.297	1.000
AIMS65 vs. MELD	283/28	0.528/0.520	+0.007 (−0.093 to +0.108)	0.885	1.000

Each pair uses the intersection of available scores. Differences are first minus second; intervals are unadjusted DeLong 95% intervals. Holm adjustment is across 15 tests within this endpoint. These revision analyses compare scores within an endpoint and do not test heterogeneity between endpoints.

**Table A2:** All 15 paired comparisons for primary haemostasis failure.

Comparison First vs. Second	*n*/Events	Paired AUROCs First/Second	Difference 95% CI	*p*	Holm *p*
Child–Pugh vs. ACLF grade	288/13	0.469/0.534	−0.066 (−0.208 to +0.077)	0.365	1.000
Child–Pugh vs. MELD 3.0	279/13	0.470/0.415	+0.055 (−0.033 to +0.143)	0.219	1.000
Child–Pugh vs. MELD-Sodium	279/13	0.470/0.405	+0.065 (−0.022 to +0.152)	0.142	1.000
Child–Pugh vs. AIMS65	288/13	0.469/0.449	+0.020 (−0.118 to +0.158)	0.779	1.000
Child–Pugh vs. MELD	283/13	0.467/0.407	+0.060 (−0.024 to +0.143)	0.162	1.000
ACLF grade vs. MELD 3.0	279/13	0.536/0.415	+0.120 (−0.038 to +0.279)	0.136	1.000
ACLF grade vs. MELD-Sodium	287/13	0.538/0.407	+0.130 (−0.034 to +0.294)	0.119	1.000
ACLF grade vs. AIMS65	292/14	0.525/0.459	+0.066 (−0.015 to +0.148)	0.112	1.000
ACLF grade vs. MELD	291/13	0.536/0.409	+0.127 (−0.034 to +0.287)	0.121	1.000
MELD 3.0 vs. MELD-Sodium	279/13	0.415/0.405	+0.010 (−0.044 to +0.064)	0.720	1.000
MELD 3.0 vs. AIMS65	279/13	0.415/0.449	−0.034 (−0.176 to +0.109)	0.643	1.000
MELD 3.0 vs. MELD	279/13	0.415/0.412	+0.003 (−0.066 to +0.073)	0.922	1.000
MELD-Sodium vs. AIMS65	279/13	0.405/0.449	−0.044 (−0.189 to +0.102)	0.557	1.000
MELD-Sodium vs. MELD	287/13	0.407/0.414	−0.006 (−0.055 to +0.042)	0.795	1.000
AIMS65 vs. MELD	283/13	0.445/0.407	+0.038 (−0.110 to +0.187)	0.612	1.000

Each pair uses the intersection of available scores. Differences are first minus second; intervals are unadjusted DeLong 95% intervals. Holm adjustment is across 15 tests within this endpoint. These revision analyses compare scores within an endpoint and do not test heterogeneity between endpoints.

**Table A3:** All 15 paired comparisons for rebleeding within 120 h.

Comparison First vs. Second	*n*/Events	Paired AUROCs First/Second	Difference 95% CI	*p*	Holm *p*
Child–Pugh vs. ACLF grade	288/16	0.673/0.654	+0.018 (−0.116 to +0.153)	0.789	1.000
Child–Pugh vs. MELD 3.0	279/16	0.674/0.651	+0.023 (−0.059 to +0.104)	0.584	1.000
Child–Pugh vs. MELD-Sodium	279/16	0.674/0.626	+0.047 (−0.038 to +0.133)	0.278	1.000
Child–Pugh vs. AIMS65	288/16	0.673/0.578	+0.095 (−0.010 to +0.199)	0.075	0.978
Child–Pugh vs. MELD	283/16	0.671/0.584	+0.088 (−0.002 to +0.177)	0.055	0.825
ACLF grade vs. MELD 3.0	279/16	0.656/0.651	+0.005 (−0.108 to +0.118)	0.934	1.000
ACLF grade vs. MELD-Sodium	287/17	0.641/0.602	+0.039 (−0.070 to +0.147)	0.484	1.000
ACLF grade vs. AIMS65	292/16	0.656/0.582	+0.074 (−0.081 to +0.229)	0.349	1.000
ACLF grade vs. MELD	291/17	0.639/0.558	+0.081 (−0.037 to +0.199)	0.178	1.000
MELD 3.0 vs. MELD-Sodium	279/16	0.651/0.626	+0.025 (−0.026 to +0.075)	0.342	1.000
MELD 3.0 vs. AIMS65	279/16	0.651/0.579	+0.072 (−0.052 to +0.195)	0.254	1.000
MELD 3.0 vs. MELD	279/16	0.651/0.588	+0.063 (−0.003 to +0.128)	0.060	0.840
MELD-Sodium vs. AIMS65	279/16	0.626/0.579	+0.047 (−0.075 to +0.169)	0.448	1.000
MELD-Sodium vs. MELD	287/17	0.602/0.562	+0.040 (−0.015 to +0.095)	0.153	1.000
AIMS65 vs. MELD	283/16	0.575/0.584	−0.008 (−0.137 to +0.120)	0.901	1.000

Each pair uses the intersection of available scores. Differences are first minus second; intervals are unadjusted DeLong 95% intervals. Holm adjustment is across 15 tests within this endpoint. These revision analyses compare scores within an endpoint and do not test heterogeneity between endpoints.

**Table A4:** Recorded and recomputed scores on identical available cases.

Score and Endpoint	*n*/Events	Recorded AUROC 95% CI	Recomputed AUROC 95% CI	Recorded Minus Recomputed 95% CI
Child–Pugh Technical failure	277/12	0.517 (0.370–0.664)	0.507 (0.365–0.649)	+0.010 (−0.011 to +0.031)
MELD-Sodium Technical failure	281/12	0.452 (0.310–0.594)	0.417 (0.258–0.575)	+0.035 (−0.038 to +0.108)
Child–Pugh Rebleeding	277/14	0.659 (0.506–0.813)	0.660 (0.508–0.811)	−0.001 (−0.029 to +0.028)
MELD-Sodium Rebleeding	281/15	0.601 (0.435–0.767)	0.586 (0.415–0.756)	+0.015 (−0.057 to +0.087)
Child–Pugh Composite	277/25	0.613 (0.502–0.725)	0.609 (0.499–0.719)	+0.004 (−0.015 to +0.024)
MELD-Sodium Composite	281/26	0.536 (0.416–0.656)	0.528 (0.405–0.651)	+0.008 (−0.034 to +0.050)

The denominator is the intersection of the recorded and recomputed versions of the same score. Intervals for differences use paired DeLong covariance. Different event counts across Table 4, Table A4 and Table A5 arise from different availability requirements. These descriptive sensitivity estimates do not test differences between component outcomes.

**Table A5:** Sensitivity comparisons against MELD after substitution of recorded scores.

Endpoint and Score Compared with MELD	*n*/Events	Paired AUROCs Score/MELD	Difference 95% CI	*p*	Holm *p*
Composite Child–Pugh (recorded)	280/26	0.597/0.495	+0.102 (+0.037 to +0.166)	0.002	0.010
Composite ACLF grade	291/29	0.607/0.508	+0.099 (−0.002 to +0.200)	0.055	0.219
Composite MELD 3.0	279/28	0.552/0.525	+0.027 (−0.020 to +0.074)	0.261	0.578
Composite MELD-Sodium (recorded)	285/26	0.532/0.496	+0.036 (−0.018 to +0.091)	0.193	0.578
Composite AIMS65	283/28	0.528/0.520	+0.007 (−0.093 to +0.108)	0.885	0.885
Technical failure Child–Pugh (recorded)	280/12	0.515/0.441	+0.075 (−0.018 to +0.168)	0.116	0.580
Technical failure ACLF grade	291/13	0.536/0.409	+0.127 (−0.034 to +0.287)	0.121	0.580
Technical failure MELD 3.0	279/13	0.415/0.412	+0.003 (−0.066 to +0.073)	0.922	1.000
Technical failure MELD-Sodium (recorded)	285/12	0.447/0.412	+0.035 (−0.048 to +0.118)	0.406	1.000
Technical failure AIMS65	283/13	0.445/0.407	+0.038 (−0.110 to +0.187)	0.612	1.000
Rebleeding Child–Pugh (recorded)	280/15	0.629/0.511	+0.118 (+0.040 to +0.195)	0.003	0.014
Rebleeding ACLF grade	291/17	0.639/0.558	+0.081 (−0.037 to +0.199)	0.178	0.470
Rebleeding MELD 3.0	279/16	0.651/0.588	+0.063 (−0.003 to +0.128)	0.060	0.240
Rebleeding MELD-Sodium (recorded)	285/15	0.598/0.536	+0.062 (−0.024 to +0.147)	0.157	0.470
Rebleeding AIMS65	283/16	0.575/0.584	−0.008 (−0.137 to +0.120)	0.901	0.901

Recorded Child–Pugh and MELD-Sodium replace their recomputed versions; the other score definitions are unchanged. Each comparison uses its own available-case intersection. Holm adjustment is across the five comparisons with MELD separately for each endpoint, not across all 15 rows. Differences in case availability as well as score values can affect comparison with the primary analysis.

**Table A6:** Descriptive findings by recorded pre-emptive TIPS status.

Characteristic or Event	Recorded Pre-Emptive TIPS	No Recorded Pre-Emptive TIPS
Admissions	63	240
Child–Pugh median (IQR), available *n*	11 (10–12), *n* = 58	9 (7–11), *n* = 230
MELD median (IQR), available *n*	21 (18–23), *n* = 60	16 (13–20), *n* = 231
Active bleeding at endoscopy	42/63 (66.7%)	78/240 (32.5%)
Portal vein thrombosis	9/63 (14.3%)	37/240 (15.4%)
Technical haemostasis failure	4/63 (6.3%)	11/240 (4.6%)
Recorded rebleeding within 120 h	2/63 (3.2%)	15/240 (6.3%)
Composite failure	6/63 (9.5%)	25/240 (10.4%)

Child–Pugh and MELD are recomputed. The comparison group includes rescue-TIPS admissions. Treatment labels are those recorded in the database; the timing and indication could not be independently adjudicated. Four admissions labelled pre-emptive TIPS also had index technical failure. Without a documented treatment chronology, this cannot be reconciled as a guideline-defined treatment sequence. No causal effect, untreated risk or treatment-specific score performance is inferred; there were only two recorded rebleeds in the pre-emptive group and ascertainment after transfer may be incomplete.

**Table A7:** Sensitivity to the overlapping admission and ligation-ulcer events.

Score	Technical Failure Overlapping Admission Excluded	Rebleeding Overlapping Admission Excluded	Rebleeding Ligation-Ulcer Events Excluded
Child–Pugh	0.489 (0.335–0.643) *n* = 287; events = 12	0.702 (0.573–0.832) *n* = 287; events = 15	0.659 (0.507–0.811) *n* = 288; events = 14
ACLF grade	0.526 (0.399–0.653) *n* = 302; events = 14	0.657 (0.517–0.796) *n* = 302; events = 16	0.601 (0.460–0.743) *n* = 303; events = 15
MELD 3.0	0.417 (0.261–0.572) *n* = 278; events = 12	0.667 (0.511–0.822) *n* = 278; events = 15	0.614 (0.453–0.776) *n* = 279; events = 14
MELD-Sodium	0.433 (0.284–0.583) *n* = 286; events = 12	0.634 (0.481–0.787) *n* = 286; events = 16	0.556 (0.392–0.721) *n* = 287; events = 15
AIMS65	0.473 (0.331–0.616) *n* = 291; events = 13	0.602 (0.473–0.731) *n* = 291; events = 15	0.568 (0.426–0.710) *n* = 292; events = 14
MELD	0.435 (0.295–0.575) *n* = 290; events = 12	0.587 (0.435–0.739) *n* = 290; events = 16	0.528 (0.364–0.692) *n* = 291; events = 15

Excluding the admission coded with both components removes that admission from both analyses. The restricted rebleeding definition excludes the two documented ligation-ulcer events but retains those admissions as non-events for that restricted endpoint; other component labels are unchanged. Values are AUROCs with unadjusted DeLong 95% intervals. These analyses do not adjudicate the underlying events or establish different mechanisms.
