# Supplementary material for: Admission Prognostic Scores and Endoscopic Findings in Relation to the Two Components of Five-Day Treatment Failure in Acute Variceal Bleeding: An Exploratory Single-Centre Retrospective Cohort Study
Source: Diagnostics (Basel). 2026 Nov 2;16(21):3620. doi: 10.3390/diagnostics16213620 (PMC13761729; doi:10.3390/diagnostics16213620)
Supplement: Supplementary file 1 [file diagnostics-16-03620-s001.zip › Table S1 STROBE Statement.pdf]

## STROBE Statement — Checklist of items that should be included in reports of cohort studies

*Manuscript: "Admission Prognostic Scores and Endoscopic Findings Show Different Associations with the Two Components of Five-Day Treatment Failure in Acute Variceal Bleeding: A Single-Centre Retrospective Cohort Study". Page numbers refer to the submitted manuscript file. Items that the manuscript does not address are stated as such rather than cross-referenced.*

| Item | Recommendation | Reported in |
|---|---|---|
| **Title and abstract** | | |
| 1 | (a) Indicate the study's design with a commonly used term in the title or the abstract | Title, p. 1 — "A Single-Centre Retrospective Cohort Study" |
| | (b) Provide in the abstract an informative and balanced summary of what was done and what was found | Abstract, p. 1; the findings are presented as exploratory |
| **Introduction** | | |
| 2 | Background/rationale: scientific background and rationale for the investigation | Section 1, pp. 2–3, paragraphs 1–5 |
| 3 | Objectives: state specific objectives, including any prespecified hypotheses | Section 1, p. 3, final paragraph. The methodological question, whether the two components of the composite have the same determinants, is posed in the third paragraph of the Introduction and is stated there to be a decomposition of an endpoint already recorded |
| **Methods** | | |
| 4 | Study design: present key elements of study design early in the paper | Section 2.1, p. 3 |
| 5 | Setting: describe the setting, locations, and relevant dates, including periods of recruitment, exposure, follow-up, and data collection | Section 2.1, p. 3 — one tertiary emergency centre, 8 January 2021 to 26 December 2025; TIPS not available on site |
| 6 | (a) Eligibility criteria, and the sources and methods of selection of participants; describe methods of follow-up | Section 2.1, p. 3. Follow-up is confined to the index admission; Section 2.2, p. 4 explains why long-term vital status could not be ascertained |
| | (b) For matched studies, give matching criteria and number of exposed and unexposed | Not applicable — unmatched cohort |
| 7 | Variables: clearly define all outcomes, exposures, predictors, potential confounders, and effect modifiers | Section 2.3, p. 4 (scores and their provenance); Section 2.4, p. 5 (the composite outcome, its two components, the secondary outcomes). The outcome is described as recorded in routine practice rather than adjudicated against written criteria |
| 8 | Data sources/measurement: for each variable of interest, give sources of data and details of methods of assessment | Section 2.2, p. 4; Section 2.3, p. 4 — five scores recomputed from their components, the ACLF grade taken as recorded; agreement of the recorded scores with recomputation is reported |
| 9 | Bias: describe any efforts to address potential sources of bias | Section 2.5, p. 5 (paired denominators, Holm adjustment, two imputation approaches); Section 3.7, pp. 15–17 (sensitivity analyses for early death and for unrecorded events after transfer); Section 4.2, p. 18 (treatment as a competing explanation); Section 4.7, pp. 20–21 |
| 10 | Study size: explain how the study size was arrived at | Not formally calculated. The cohort was fixed by the inclusion period (Section 2.1, p. 3); events per variable in Section 3.7, p. 15; the limitation in power in Section 4.7, p. 20 |
| 11 | Quantitative variables: explain how quantitative variables were handled; describe which groupings were chosen and why | Section 2.3, p. 4 (scores on their continuous scales; Child–Pugh classes derived from the recomputed score); Table 5, p. 14 (thresholds) |
| 12 | (a) Describe all statistical methods, including those used to control for confounding | Section 2.5, p. 5, with software and package versions; the full analysis code is provided as Supplementary Material |
| | (b) Describe any methods used to examine subgroups and interactions | Section 2.4, p. 5 — the component analysis was specified before any model was fitted; results in Section 3.5, pp. 12–13 |
| | (c) Explain how missing data were addressed | Section 2.2, p. 4 and Section 2.5, p. 5 — complete-case analysis, multiple imputation with thresholding of binary variables and, as a sensitivity analysis, type-matched chained equations with logistic imputation of binary variables; results in Section 3.7 and Table 7, p. 17 |
| | (d) If applicable, explain how loss to follow-up was addressed | A five-day outcome is recorded for every admission; Section 3.1, p. 7 states that a recorded value is not proof of complete ascertainment, and Section 3.7 explores |

| | | |
|---|---|---|
| | | unrecorded events after transfer by simulation. Mortality was not analysed (Section 2.2, p. 4) |
| | (e) Describe any sensitivity analyses | Section 3.7, pp. 15–17: two imputation approaches, death within 120 hours added to the composite, and 2,000 simulations of unrecorded rebleeds among the transferred admissions |
| **Results** | | |
| 13 | (a) Report numbers of individuals at each stage of the study | Section 3.1, p. 7; Figure 1, p. 7 |
| | (b) Give reasons for non-participation at each stage | Section 3.1, p. 7. No admission was excluded from the primary analysis; the model set of 288 excludes 15 admissions with a missing model variable |
| | (c) Consider use of a flow diagram | Figure 1, p. 7 |
| 14 | (a) Give characteristics of study participants and information on exposures and potential confounders | Table 1, p. 8; Section 3.1, p. 7 |
| | (b) Indicate number of participants with missing data for each variable of interest | Table 1, p. 8, "Missing" column; Tables 3 and 4, p. 10; Table 5, p. 14 |
| | (c) Summarise follow-up time | Not applicable beyond the index admission (Section 2.2, p. 4) |
| 15 | Outcome data: report numbers of outcome events or summary measures over time | Section 3.1, p. 7 (31 events: 15 failures of primary haemostasis and 17 rebleeds); Table 2, p. 9 |
| 16 | (a) Give unadjusted estimates and, if applicable, confounder-adjusted estimates and their precision | Table 6, p. 16; Sections 3.4 and 3.7 |
| | (b) Report category boundaries when continuous variables were categorised | Table 5, p. 14 |
| | (c) If relevant, consider translating estimates of relative risk into absolute risk | Figure 5, p. 14 (observed failure by stratum); Section 3.7 (net benefit) |
| 17 | Other analyses: report other analyses done | Section 3.4, p. 11 (red wale markings); Section 3.5, pp. 12–13 (component analysis); Section 3.7, pp. 15–17 (internal validation and sensitivity analyses) |
| **Discussion** | | |
| 18 | Key results: summarise key results with reference to study objectives | Section 4, p. 17, opening paragraph |
| 19 | Limitations: discuss limitations, taking into account sources of potential bias or imprecision | Section 4.7, pp. 20–21: power, conditioning on ligation, absence of blinded re-review, retrospective outcome criteria, ascertainment after transfer, data quality of the recorded scores, the record identifier, absence of HVPG |
| 20 | Interpretation: give a cautious overall interpretation considering objectives, limitations, multiplicity of analyses, results from similar studies, and other relevant evidence | Sections 4.1 to 4.6, pp. 17–20; Section 5, p. 21 |
| 21 | Generalisability: discuss the generalisability (external validity) of the study results | Section 4.7, p. 20; Section 4.5, p. 19 |
| **Other information** | | |
| 22 | Funding: give the source of funding and the role of the funders for the present study | Funding statement, p. 22 — no external funding |

*An Explanation and Elaboration article discusses each checklist item and gives methodological background and published examples of transparent reporting. The STROBE checklist is best used in conjunction with this article (freely available at www.equator-network.org).*
