# Supplementary material for: Admission Prognostic Scores and Endoscopic Findings in Relation to the Two Components of Five-Day Treatment Failure in Acute Variceal Bleeding: An Exploratory Single-Centre Retrospective Cohort Study
Source: Diagnostics (Basel). 2026 Nov 2;16(21):3620. doi: 10.3390/diagnostics16213620 (PMC13761729; doi:10.3390/diagnostics16213620)
Supplement: Supplementary file 1 [file diagnostics-16-03620-s001.zip › Table S2 TRIPOD Checklist Prediction Model Development.pdf]

TRIPOD Checklist: Prediction Model Development

Manuscript: "Admission Prognostic Scores and Endoscopic Findings Show Different Associations with the Two Components of Five-Day Treatment Failure in Acute Variceal Bleeding: A Single-Centre Retrospective Cohort Study". Page numbers refer to the submitted manuscript file. Items that the manuscript does not address are stated as such rather than cross-referenced. This is a model development study without external validation; items that apply only to validation are recorded as not applicable.

Section/Topic	Item	Checklist Item	Reported in
**Title and Abstract**			
Title	1	Identify the study as developing and/or validating a multivariable prediction model, the target population, and the outcome to be predicted	p. 1
Abstract	2	Provide a summary of objectives, study design, setting, participants, sample size, predictors, outcome, statistical analysis, results, and conclusions	p. 1
**Introduction**			
Background and objectives	3a	Explain the medical context and rationale for developing or validating the model, including references to existing models	Section 1, pp. 2–3
	3b	Specify the objectives, including whether the study describes the development or validation of the model or both	Section 1, p. 3 — development only
**Methods**			
Source of data	4a	Describe the study design or source of data	Section 2.1, p. 3 — retrospective cohort, single data set
	4b	Specify the key study dates, including start of accrual, end of accrual, and, if applicable, end of follow-up	Section 2.1, p. 3; follow-up confined to the index admission (Section 2.2, p. 4)
Participants	5a	Specify key elements of the study setting including number and location of centres	Section 2.1, p. 3 — one tertiary emergency centre
	5b	Describe eligibility criteria for participants	Section 2.1, p. 3
	5c	Give details of treatments received, if relevant	Section 3.2, p. 9; Table 2, p. 9; Section 4.6, p. 19 (recorded eligibility for pre-emptive TIPS against treatment received)
Outcome	6a	Clearly define the outcome that is predicted by the prediction model, including how and when assessed	Section 2.4, p. 5 — treatment failure within 120 hours comprising failure to control bleeding at the index endoscopy and rebleeding, each also analysed separately. The detailed criteria were those of routine practice, not recorded in a written protocol, and the outcome is described as recorded rather than adjudicated; stated in Section 2.4 and Section 4.7
	6b	Report any actions to blind assessment of the outcome to be predicted	Not reported. Outcomes were abstracted retrospectively and no blinding to predictor values was possible (Section 4.7, p. 20)
Predictors	7a	Clearly define all predictors used in developing the multivariable prediction model, including how and when they were measured	Section 2.2, p. 4; Section 2.3, p. 4
	7b	Report any actions to blind assessment of predictors for the outcome and other predictors	Not reported. Endoscopic findings were abstracted from routine reports without standardised re-review (Section 4.7, p. 20)
Sample size	8	Explain how the study size was arrived at	Not formally calculated; events per variable in Section 3.7, p. 15; limitation in Section 4.7, p. 20
Missing data	9	Describe how missing data were handled, with details of any imputation method	Section 2.5, p. 5 — complete-case analysis; multiple imputation by chained equations with 25

			imputations, continuous variables by Bayesian ridge regression with posterior sampling and binary variables restored by thresholding; as a sensitivity analysis, type-matched chained equations with logistic imputation of binary variables; pooling by Rubin's rules. Results in Section 3.7 and Table 7, p. 17; implementation in the Supplementary Material
Statistical analysis methods	10a	Describe how predictors were handled in the analyses	Section 2.3, p. 4; Section 2.5, p. 5
	10b	Specify type of model, all model-building procedures (including any predictor selection), and method for internal validation	Section 2.5, p. 5 — logistic regression limited to three pre-specified variables; bootstrap validation with 1,000 replicates and refitting within each replicate
	10c	For validation, describe how the predictions were calculated	Not applicable — development only
	10d	Specify all measures used to assess model performance and, if relevant, to compare multiple models	Section 2.5, p. 5 — AUROC with DeLong intervals and Holm adjustment, likelihood-ratio tests for nested models, optimism-corrected calibration slope, decision curve analysis
	10e	Describe any model updating arising from the validation	Not applicable — development only
Risk groups	11	Provide details on how risk groups were created, if done	No risk groups were derived; event rates by class and grade are descriptive (Section 3.6, p. 13; Figure 5, p. 14)
Development vs. validation	12	For validation, identify any differences from the development data	Not applicable — development only
**Results**			
Participants	13a	Describe the flow of participants, including the number with and without the outcome and a summary of follow-up time	Section 3.1, p. 7; Figure 1, p. 7
	13b	Describe the characteristics of the participants, including the number with missing data for predictors and outcome	Table 1, p. 8
	13c	For validation, show a comparison with the development data	Not applicable — development only
Model development	14a	Specify the number of participants and outcome events in each analysis	Section 3.7, p. 15 (288 admissions, 28 events); Tables 3, 4 and 5 give the denominator and events of every other analysis
	14b	If done, report the unadjusted association between each candidate predictor and outcome	Table 6, p. 16
Model specification	15a	Present the full prediction model to allow predictions for individuals	Table 6, p. 16 gives the adjusted odds ratios; the intercept is not presented and the manuscript does not propose the model for clinical use (Sections 4.4 and 5). The fitted model can be reproduced exactly from the Supplementary Material
	15b	Explain how to use the prediction model	Not applicable in the intended sense; the model is not proposed for use
Model performance	16	Report performance measures (with CIs) for the prediction model	Section 3.7, p. 15; Figure 6, p. 17
Model updating	17	If done, report the results from any model updating	Not applicable — development only
**Discussion**			
Limitations	18	Discuss any limitations of the study	Section 4.7, pp. 20–21
Interpretation	19a	For validation, discuss the results with reference to performance in the development data	Not applicable — development only

	19b	Give an overall interpretation of the results	Sections 4.1 to 4.6, pp. 17–20; Section 5, p. 21
Implications	20	Discuss the potential clinical use of the model and implications for future research	Section 4.6, p. 19; Section 5, p. 21
**Other information**			
Supplementary information	21	Provide information about the availability of supplementary resources	Data Availability Statement, p. 22 — the analysis code that reproduces every result is provided as Supplementary Material; the dataset is available on reasonable request
Funding	22	Give the source of funding and the role of the funders for the present study	Funding statement, p. 22 — no external funding

We recommend using the TRIPOD Checklist in conjunction with the TRIPOD Explanation and Elaboration document.
